# Persistence of memory: lifespan dynamics of the human antiviral antibody reactome

**DOI:** 10.1038/s41467-026-74680-y

**Published:** 2026-06-22

**Authors:** Moriah M. Mitchell, Tomasz Kula, Jennifer L. Remmel, Siyang Xia, Ellen L. Shrock, Yumei Leng, H. Benjamin Larman, Yu Chen, Mikael Knip, Marianna Cortese, Michael J. Mina, Alberto Ascherio, Stephen J. Elledge

**Affiliations:** 1 Division of Genetics, Department of Medicine, Howard Hughes Medical Institute, Brigham and Women’s Hospital, Boston, MA USA; 2 Department of Genetics, Harvard Medical School, Boston, MA USA; 3 Program in Systems, Synthetic, and Quantitative Biology, Harvard University, Boston, MA USA; 4 Center for Communicable Disease Dynamics, Harvard T.H. Chan School of Public Health, Boston, MA USA; 5 Department of Epidemiology, Harvard T.H. Chan School of Public Health, Boston, MA USA; 6 Department of Pathology, Johns Hopkins University School of Medicine, Baltimore, MD USA; 7 Institute for Cell Engineering, Johns Hopkins University School of Medicine, Baltimore, MD USA; 8 Department of Population Health, NYU Grossman School of Medicine, New York, NY USA; 9 Research Program for Clinical and Molecular Metabolism, Faculty of Medicine, University of Helsinki, Helsinki, Finland; 10 Turku Bioscience Centre, University of Turku and Åbo Akademi University, Turku, Finland; 11 Department of Nutrition, Harvard T.H. Chan School of Public Health, Boston, MA USA; 12 Department of Immunology and Infectious Diseases, Harvard T.H. Chan School of Public Health, Boston, MA USA; 13 Department of Pathology, Brigham and Women’s Hospital, Boston, MA USA; 14 Channing Division of Network Medicine, Department of Medicine, Brigham and Women’s Hospital and Harvard Medical School, Boston, MA USA; 15 Present Address: Generate: Biomedicines, Somerville, MA USA; 16 Present Address: Intellia Therapeutics, Cambridge, MA USA

**Keywords:** Infectious diseases, Adaptive immunity

## Abstract

The human antiviral antibody reactome provides a cumulative molecular record of immune exposures. Using high-resolution VirScan profiling, we compared epitope-level antibody responses across early childhood and adulthood. Infants are born with maternal IgG antibodies, but these antibodies decay rapidly and are replaced by endogenous responses to ~22 new viral exposures within three years. Pediatric antibody reactivities remain highly dynamic until about age 7 and are broad in epitope specificity but largely short-lived. In contrast, adult reactomes are remarkably stable and individualized, enabling accurate longitudinal donor identification (Immunoprint, > 99.99% accuracy). Stability varies by viral family, with *Pneumoviridae* and *Picornaviridae* persisting more robustly than *Coronaviridae* or *Orthomyxoviridae*. Across ages, immunodominant epitopes and initial binding strength predict response persistence. Longitudinal profiling highlights biological and epidemiological drivers of reactome change. This population-level, age-stratified atlas informs our understanding of immune memory and development with applications to vaccine design, surveillance, and precision public health.

## Abstract (web summary)

This study shows how antiviral antibody profiles evolve from infancy to adulthood, with rapid remodeling in early life and stable, individual immune histories in adults, revealing the number of viral infections infants experience each year.

## Introduction

The antibody repertoire, i.e. the complete collection of antibodies an individual produces, provides a molecular record of immune exposures acquired through infection and vaccination. This record reflects how the immune system protects throughout life; however, studying it remains challenging. B cell receptor (BCR) sequencing does not directly report functional epitope specificities, and traditional binding assays lack epitope-level resolution across viral proteomes. Despite these challenges, advancing our understanding of population-level diversity and persistence of the antiviral antibody responses is essential for informing immunization strategies and safeguarding population immunity.

Our current understanding of population-level antibody diversity is largely based on BCR sequencing or pathogen-specific analysis of antibody binding. Analysis of BCR sequences from limited numbers of donors has revealed both striking inter-individual diversity and shared features^1^. Pathogen-specific analyses of antibody responses show differences in epitope targeting associated with demographic and disease-related factors^2,3^, and both genetic and environmental determinants of antibody responses to microbial antigens have been reported^4,5^. At the same time, different individuals often mount remarkably similar antibody responses to the same virus^1,6^, recognizing common “public epitopes”. Nevertheless, a more comprehensive understanding of population-level diversity of antiviral antibody responses and the mechanisms underlying both common and rare patterns of epitope targeting is needed.

Studies of antibody titers to selected vaccine antigens suggest remarkable long-term stability in adults^7,8^, but whether this stability extends to the level of epitope-specific responses or across the breadth of the human virome remains unclear. Overall, antibody responses to certain microbiota appear relatively stable in adults^4^; however, systematic, high-resolution analyses are needed to determine whether this pattern holds for antibodies to diverse viruses and whether there are shared patterns of peptide-specific circulating antibody persistence across individuals. Such insights would be particularly valuable for understanding how antibody stability and diversity may change over the lifespan. Although many studies have documented differences in antibody responses between children and adults at single time points, longitudinal studies are sparse^3,5,9–12^. Though the greatest shifts in antiviral immunity are thought to occur early in life and again in old age^13,14^, little is known about how the stability of antibody responses evolves from early childhood to adulthood.

To address these questions, we focused on the antiviral antibody reactome (AAR), which we define here as the viral peptide specificities of serum antibodies measured using VirScan. Unlike the broader antibody repertoire, which encompasses all underlying B cell clones, the AAR is tractable at scale and directly reveals which viral epitopes have been targeted over an individual’s exposure history. We measured antiviral antibody specificities in large longitudinal and cross-sectional cohorts, using VirScan to explore the diversity and dynamics of the AAR in early childhood and adulthood. We compared antiviral responses between adults and children, characterized AAR stability and diversity, and examined the development of antiviral immunity in early life. We found two striking differences between adult and pediatric reactomes: pediatric reactomes are dynamic yet exhibit broad antiviral specificity, whereas adult reactomes are remarkably durable but have narrower specificity. We harness the stability of the adult AAR to present a novel identity-matching tool, Immunoprint, that links longitudinal samples with >99.99% precision. In contrast, we systematically characterize the rapid turnover of the antibody reactome in early life, driven by maternal antibody decay and exposure to more than 20 viruses in the first three years of life. We identify viral family-specific reactome stability and demonstrate that antibody responses targeting “public epitopes” and those with higher initial levels are more likely to persist. These insights offer actionable guidance for deploying epitope-resolved serology as a public health resource and designing age-tailored immunogens.

## Results

### Early endogenous antiviral reactome composition differs from adult reactome composition at the population level

**Fig. 1:**
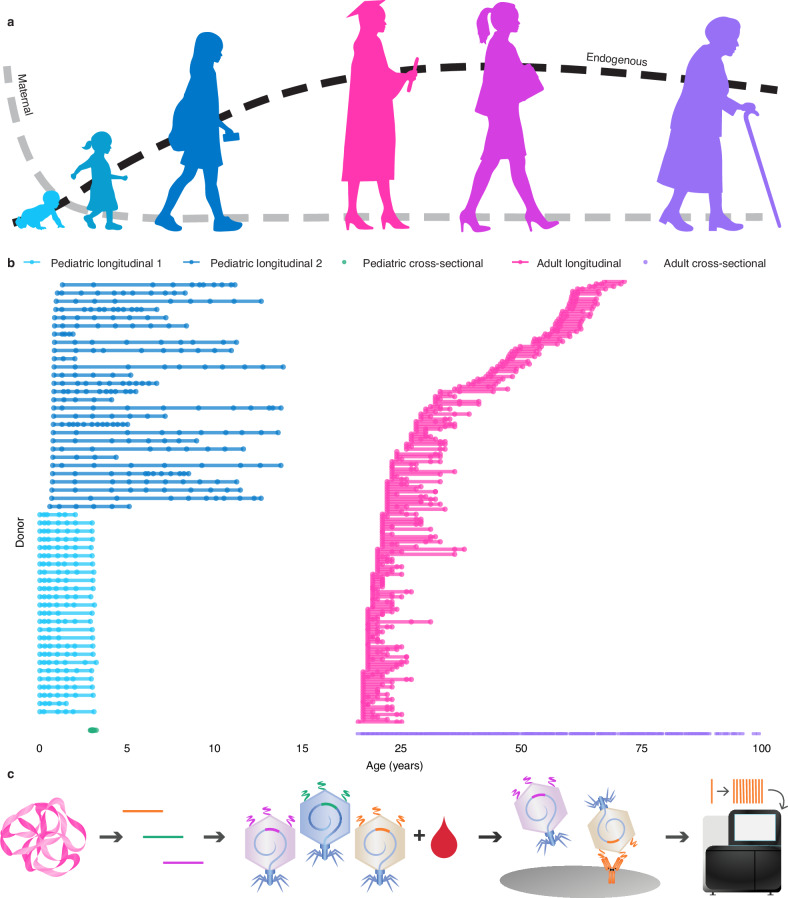
Study and data set overview. **a** Schematic of maternal antibody waning and endogenous antibody reactome development over the course of a lifetime. **b** Summary of age, number of donors, and sampling intervals for longitudinal donors by data set (color). Longitudinal samples from the same donor are connected by lines. **c** VirScan method, drawn by the authors and adapted from Xu et al.^9^. The library was designed by tiling the proteomes of selected viruses into 56 amino acid fragments that were cloned into a T7 phage vector. The library is incubated with human sera. If an antibody recognizes a peptide displayed in the library, it will bind and be immunoprecipitated while unbound phage is washed away. Phage insert DNA is PCR amplified, barcoded, and sequenced.

We compared early-life AARs with those of adults to determine how pediatric profiles relate to adult profiles for given viral exposures (Fig. 1). Using VirScan, we profiled epitope specificities in the Adult Cross-Sectional Cohort and Pediatric Longitudinal Cohort 1 (Table 1, Table 2, Supplementary Data 1–4).

**Table 1:** Summary of cohorts from which serum samples were obtained

Cohort	Number of donors	Number of samples	Age group	Longitudinal	Age known	Sex known	Collection years	Country
C1	392	392	Adult	No	Yes	Yes	2020	United States
C2	40	116	Adult	Yes	Yes	Yes	1985–1997	United States
C3	167	391	Adult	Yes	Yes	Yes	1993–2013	United States
C4	30	187	Pediatric	Yes	Yes	Yes	2008–2012	Finland
C5	28	254	Pediatric	Yes	Yes	Yes	2004–2010	United States

**Table 2:** Summary of data set demographics

Data set	Cohorts represented	Number of samples	Number of donors	Donor sex distribution (% Female)	Age range (years)
Pediatric cross-sectional	C5	27	27	41	2.8–3.2
Pediatric longitudinal 1	C4	160	25	44	0–3.2
Pediatric longitudinal 2	C5	254	28	43	0.5–14
Adult cross-sectional	C1, C3	559	559	38	16–99
Adult longitudinal	C2, C3	477	177	27	16–71

**Fig. 2:**
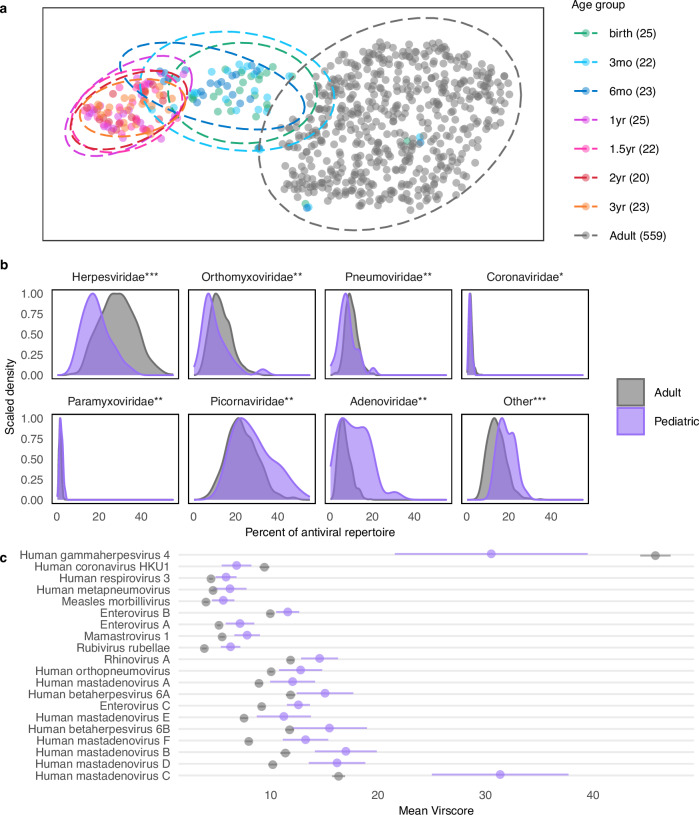
Composition and breadth of the early endogenous reactome differ from those of the adult reactome. **a** Output of UMAP dimension reduction plot generated using hit data for all library peptides (n_neighbors = 15, min_dist=0.1, metric = euclidean). Sample size for each age group is included in parentheses in the legend. Each dot is colored according to age of the donor at time of sample collection. Ellipses indicate the 95% confidence region for each age group, estimated using a multivariate t-distribution. **b** Distributions of percent reactome composition by viral family according to age group, based on 559 adult and 27 pediatric (age 2.5–3.5 years) samples from distinct donors. Statistical significance was assessed using two-sided Wilcoxon rank-sum tests comparing adults and children for each viral family. *P* values were adjusted for multiple comparisons using the Benjamini–Hochberg method; adjusted *P* values are annotated as *P < 0.05, **P < 0.01 and ***P < 0.001. Exact adjusted *P* values were: Herpesviridae, *P* = 2.67 × 10⁻⁸; Orthomyxoviridae, *P* = 0.00155; Pneumoviridae, *P* = 0.00155; Coronaviridae, *P* = 0.0458; Paramyxoviridae, *P* = 0.00136; Picornaviridae, *P* = 0.00691; Adenoviridae, *P* = 0.00155; Other, *P* = 8.79 × 10⁻⁷. **c** Mean VirScore ±95% confidence interval (calculated using the *t* critical value and SEM) among donors with inferred exposure, stratified by age group, for viral species exhibiting statistically significant differences in mean VirScore between children and adults. Biological replicates are distinct donors. The total datasets included 559 adult and 27 pediatric donors aged 2.5–3.5 years; virus-specific sample sizes reflect the number of donors with inferred exposure to each virus. Adult and pediatric sample sizes, respectively, were: Human mastadenovirus C, adult *n* = 549, pediatric *n* = 22; Human mastadenovirus D, adult *n* = 450, pediatric *n* = 22; Human mastadenovirus B, adult *n* = 484, pediatric *n* = 19; Human mastadenovirus F, adult *n* = 501, pediatric *n* = 20; Human betaherpesvirus 6B, adult *n* = 535, pediatric *n* = 19; Human mastadenovirus E, adult *n* = 414, pediatric *n* = 22; Enterovirus C, adult *n *= 530, pediatric *n* = 12; Human betaherpesvirus 6A, adult *n* = 482, pediatric *n* = 16; Human mastadenovirus A, adult *n* = 459, pediatric *n* = 23; Human orthopneumovirus, adult *n* = 558, pediatric *n* = 19; Rhinovirus A, adult *n* = 559, pediatric *n* = 27; Rubivirus rubellae, adult *n* = 226, pediatric *n* = 22; Mamastrovirus 1, adult *n* = 309, pediatric *n* = 16; Enterovirus A, adult *n* = 518, pediatric *n* = 20; Enterovirus B, adult *n* = 558, pediatric *n* = 27; Measles morbillivirus, adult *n* = 211, pediatric *n* = 22; Human metapneumovirus, adult *n* = 310, pediatric *n* = 14; Human respirovirus 3, adult *n* = 462, pediatric *n* = 20; Human coronavirus HKU1, adult *n* = 248, pediatric *n* = 12; Human gammaherpesvirus 4, adult *n* = 550, pediatric *n *= 15. Statistical significance was assessed using two-sided Wilcoxon rank-sum tests comparing adults and children for each virus, followed by Benjamini–Hochberg correction for multiple comparisons. Viral species shown are those with Benjamini–Hochberg-adjusted *P* < 0.05.

Dimensionality reduction with Uniform Manifold Approximation and Projection (UMAP)^15,16^ applied to binary VirScan hit data revealed progressive separation of pediatric samples from the adult samples between birth and three years (Fig. 2a). This age-associated structure was observed both across the full peptide library and when analyses were stratified by viral family (Supplementary Fig. 1), indicating that divergence is not driven by a single viral group. The pattern was robust across random seeds, hyperparameter settings, and class-balanced analyses (Supplementary Fig. 2a–c). Consistent separation was also observed in the original high-dimensional space by PERMANOVA on Jaccard distances, with significance exceeding that expected under label permutation (Supplementary Fig. 2d). Together, these analyses indicate that pediatric VirScan immunoprofiles diverge systematically from adult profiles across early life.

We next examined whether these differences arise from differential viral exposure or from heterogeneity in immune responses to the same pathogens by analyzing the Adult Cross-Sectional (*n* = 559) and Pediatric Cross-Sectional (*n* = 27) datasets. Using a two-sided Wilcoxon Rank Sum Test, we detected statistically significant differences in the proportional composition of responses devoted to seven viral families and all other viruses combined (Fig. 2b). These remained significant after Benjamini-Hochberg correction (false discovery rate (FDR) = 0.05). Adults showed a greater burden of *Herpesviridae* and *Orthomyxoviridae* responses, even in a class-balanced analysis (Supplementary Fig. 2e). In contrast, young children showed proportionally greater responses to *Picornaviridae* and *Adenoviridae*, consistent with recent exposures to rhinovirus, enterovirus, and adenovirus^17^ (Fig. 2b), though these differences failed to achieve statistical significance after downsampling the adult dataset to the size of the pediatric data set (Supplementary Fig. 2e).

### Children produce antibodies to a broader range of viral epitopes than adults do

We next examined whether the breadth of immune response, defined as the number of distinct peptide reactivities, differs between adults and three-year-old children using the adult and pediatric cross-sectional datasets. We quantified the number of distinct epitopes targeted per virus using VirScores^9^ (Supplementary Data 5) in people with inferred exposure to each virus (see Methods) and evaluated differences between children and adults using two-sided Wilcoxon Rank Sum Tests with a Benjamini-Hochberg correction for multiple hypothesis testing. We observed that exposed children target more distinct epitopes than exposed adults for most viruses (Fig. 2c). This applied to all viruses with a statistically significant difference in number of distinct peptides targeted given viral exposure except for *human gammaherpesvirus 4* and *human coronavirus HKU1* where adult breadth is higher (Fig. 2c). Many of these differences persisted in class-balanced analysis with smaller sample size (Supplementary Fig. 2f).

While these differences may in part reflect more recent primary infection in children, they may also arise from qualitative differences in the developing humoral immune response. The antibody repertoire is characterized by lower levels of somatic hypermutation and ongoing affinity maturation relative to adults^18–21^ and is enriched for polyreactive specificities^19,22^. Such features could contribute to broader peptide reactivity profiles in young children, either through reduced affinity maturation constraints or increased polyreactivity, which may inflate apparent epitope breadth. In addition, early-life immunity is supported by distinct isotype contributions, including IgM and mucosal IgA, as well as maternally derived antibodies, which together shape antigen exposure and subsequent B cell responses^23–25^. These factors may collectively influence the observed differences in epitope breadth between children and adults.

### The antibody adult reactome is relatively stable within and distinct between individuals

**Fig. 3:**
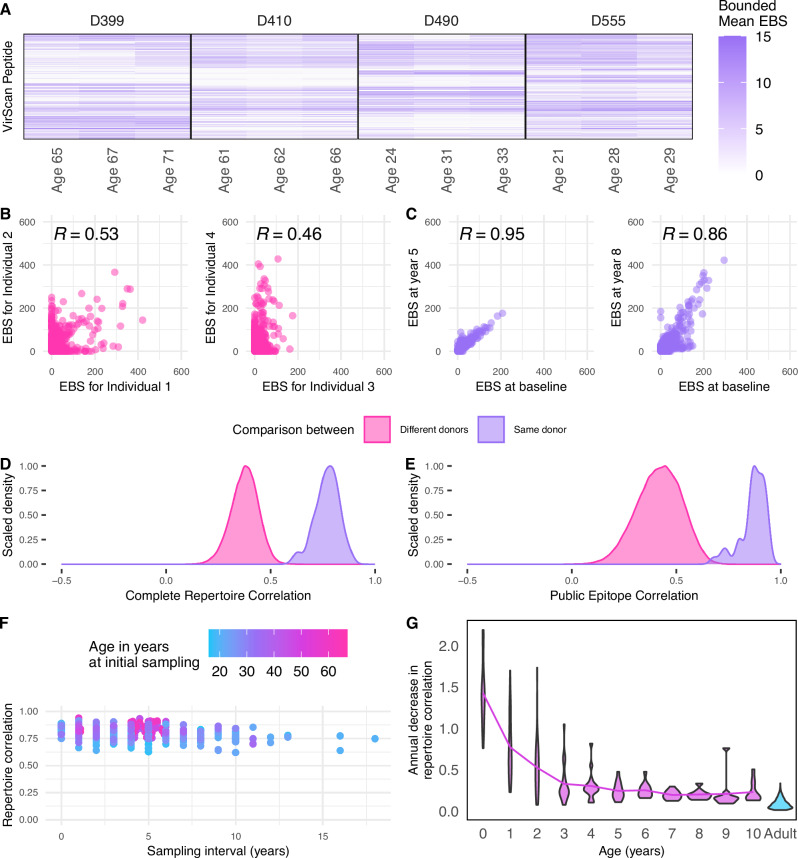
The antiviral antibody reactome is distinct between individuals and stable within adults for at least a decade. **A** Epitope binding signals for three longitudinal samples (collected at baseline, and two subsequent time points) from each of 4 donors. Peptides targeted by at least two samples are included. **B** Epitope binding signals and correlation coefficients for comparisons between longitudinal samples between two donors or (**C**) from a single donor. Distributions of within vs between individual bounded epitope binding signal correlations based on 567 samples from 431 distinct donors. For density comparisons of within- versus between-individual correlations, within-donor pairs ≤5 years apart were excluded to avoid over-representation of short-interval comparisons. Correlation coefficients were computed over the complete (**D**) and public epitope reactomes (**E**). **F** Sampling interval versus Pearson correlation in epitope binding signal for pairs of longitudinal samples from adults colored according to age at time of initial sample collection. **G** Violin plot of annual decrease in reactome correlation calculated using consecutive longitudinal samples according to age in children (magenta) compared to adults (blue) based on 25, 53, 41, 21, 16, 14, 11, 13, 9, 10, and 264 pairs of consecutive samples from 0 years of age to adults, respectively.

To assess inter-individual diversity and intra-individual stability of the adult AAR, we analyzed the VirScan epitope binding signals (EBS) of 177 adults with repeated sampling (Adult Longitudinal dataset). EBS values measure relative enrichment of antibodies to each peptide in the VirScan library (see Methods) and correlate with antibody titer^26^. Peptide-specific responses appeared qualitatively stable within individuals and distinct between individuals. Side-by-side visualizations of EBS values produced barcode-like patterns (Fig. 3A). underscoring both individual diversity and the temporal stability of the antibody reactome.

Quantitatively, EBS values from unrelated donors were weakly correlated (Fig. 3B), while serial samples of the same donor showed strong linear correlation (Fig. 3C). This finding motivated us to use correlation structure to ascertain donor identity. Using the full VirScan library maximized discrimination between inter- and intra-donor comparisons (Fig. 3D). Remarkably, even restricting analysis to 268 peptides containing previously characterized public epitopes^6^ was sufficient to differentiate inter- and intra-individual comparisons (Fig. 3E). Importantly, this stability extends across years, with high EBS auto-correlation observed even in samples collected more than fifteen years apart (Fig. 3F).

These properties enabled the development of Immunoprint (see Methods), a bioinformatic method with applications to laboratory quality control and serological surveillance. Immunoprint identifies serial samples from the same donor using EBS correlations alone, achieving >99.99% accuracy (Supplementary Fig. 3). This performance highlights the remarkable stability and individuality of the adult AAR.

### Antibody reactome stability varies by viral family, age, and strength of initial response

In contrast to the stable adult reactome, the pediatric reactome is highly dynamic. Using data from all three longitudinal datasets, we quantified the annual rate of reactome change as (1-autocorrelation)/(sampling interval in years), where autocorrelation is defined as the Pearson correlation of bounded EBS between consecutive samples from a donor. We observe rapid rates of change early in life (Fig. 3G) with the highest rates of change (1.3) between birth and one year of age, consistent with maternal antibody waning. Rates of change remained high in the second (0.7) and third (0.5) years of life as children mount their own antibody responses to vaccinations and natural infections. These rates remain elevated compared to adults but begin to approach adult-like stability (rate ~0.09) by age 7.

**Fig. 4:**
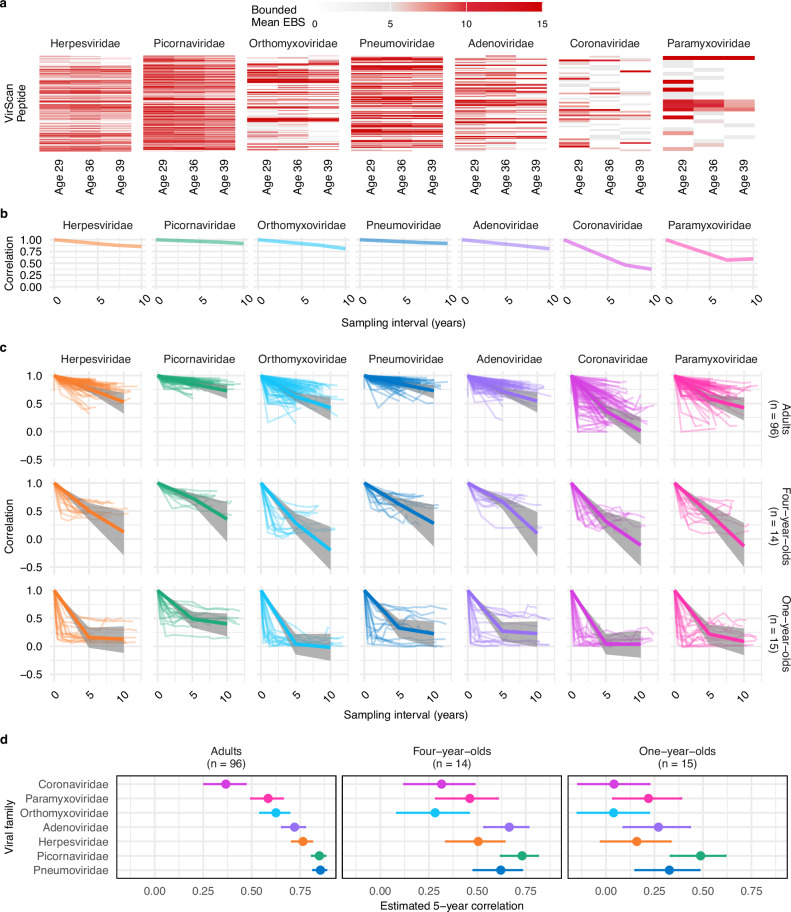
Longitudinal stability of the antibody reactome varies according to viral family and age. **a** Heatmap of epitope binding signal for peptides by viral family for adult representative donor over 10 years. Peptides displayed were restricted to those with EBS ≥ 3.5 for at least one time point to reduce sparseness. **b** Correlation in family-specific EBS relative to baseline by viral family for the same representative donor. **c** Correlation of family-specific bounded epitope binding signals (EBS) relative to baseline is shown as a function of sampling interval for adult longitudinal donors and pediatric donors beginning at 1 year or four years of age. Thin lines represent individual donor trajectories. Thick lines indicate model-estimated mean correlations from a mixed-effects model fit to Fisher z-transformed correlations using a natural spline for sampling interval and a random intercept for donor; shaded regions indicate model-derived 95% confidence intervals calculated on the Fisher z-transformed scale and back-transformed to the correlation scale. Biological replicates are distinct donors, with sample sizes indicated in the facet labels. **d** Model-estimated correlations between antibody repertoires measured 5 years apart are shown for each viral family, stratified by cohort (facets). Points indicate estimated mean five-year correlations and error bars represent model-derived 95% confidence intervals calculated on the Fisher z-transformed scale and back-transformed to the correlation scale. Biological replicates are distinct donors, with sample sizes indicated in the facet labels.

After establishing the overall stability of the adult antibody reactome, we asked whether stability varied across the *Herpesviridae*, *Picornaviridae*, *Orthomyxoviridae*, *Pneumoviridae*, *Adenoviridae*, *Coronaviridae*, and *Paramyxoviridae* families. Within-donor auto-correlation in VirScan EBS did not decrease uniformly over time across viral families, both in a representative adult and in aggregate analyses (Fig. 4a–c). Extending this analysis to the Pediatric Longitudinal 2 dataset, beginning at ages one and four, revealed lower autocorrelations in children, consistent with more dynamic antibody responses early in life. However, some patterns of relative stability were conserved across age groups: *Pneumoviridae* and *Picornaviridae* auto-correlations were among the most stable, while *Coronaviridae*, *Paramyxoviridae*, and *Orthomyxoviridae* responses were less stable(Fig. 4c, d). These differences were not attributable to unequal peptide representation in the VirScan library, as iterative downsampling analyses confirmed the robustness of these family-level patterns (Supplementary Fig. 4).

Notably, *Picornaviridae* responses were among the most stable across all age groups, despite the high genetic diversity and frequent exposure associated with this family. Several mechanisms may account for this observation. First, repeated infections with common picornaviruses, such as rhinoviruses and enteroviruses, may preferentially boost pre-existing antibody specificities rather than generate entirely novel reactomes, resulting in stable longitudinal reactivity profiles^27,28^. Second, vaccination against poliovirus and hepatitis A, both picornaviruses with high vaccination coverage in these populations, elicits highly durable neutralizing antibody responses that can persist for decades, contributing to stable serological signatures^7,8^. Finally, because VirScan measures reactivity to linear epitopes^9^, including many non-neutralizing regions, repeated exposures to antigenically drifted viruses may still boost conserved or cross-reactive epitopes, even in the context of viral evolution. These factors together may reconcile the apparent paradox of high viral diversity and exposure frequency with stable antibody reactivity profiles.

To investigate other factors underlying differences in stability, we next examined the relationship between the initial EBS magnitude and the long-term persistence of epitope binding. Virus-specific EBS values, which correlate with virus-specific antibody titers^26^, showed a strong positive association between initial EBS and final EBS values (Supplementary Fig. 5a). Logistic regression modeling demonstrated that the probability of sustained peptide recognition increased monotonically with initial EBS, approaching saturation at high values (AUC = 0.8763, Supplementary Fig. 5b). Peptides containing previously defined public epitopes were also significantly more likely to be recognized longitudinally in both adults and 1-year-olds, even after controlling for initial EBS (adjusted odds ratio = 2.32; Supplementary Fig. 5c). Together, these findings indicate that both the magnitude and immunodominance of the initial response are key determinants of antibody durability.

Overall, these findings demonstrate that antibody response stability is not uniform across viral families but instead reflects a combination of initial response magnitude, immunodominance, exposure history, and virus-specific biological features.

### The antiviral antibody reactome is exceptionally dynamic in early life

Maternal IgG antibodies transferred across the placenta wane over the first 6–12 months of life^29,30^, during which neonates have a limited capacity to mount protective B-cell response due to immune immaturity^29,30^. As children encounter antigens through vaccinations and microbial exposures in early life, endogenous B-cell responses emerge. We investigated reactome dynamics during this developmental window using the Pediatric Longitudinal 1 dataset.

**Fig. 5:**
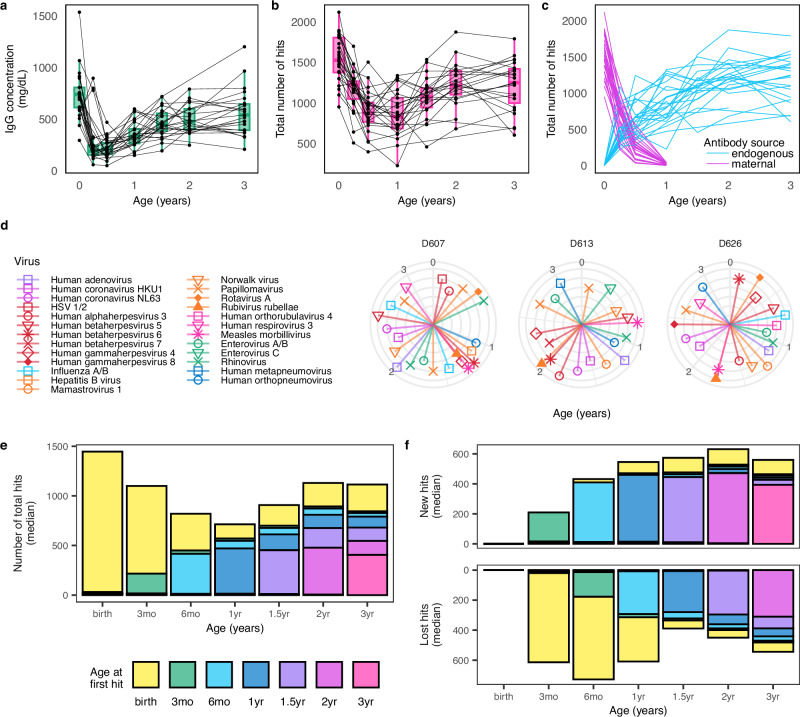
Longitudinal stability of the antibody reactome in early childhood. Data in (**a–c,** **e**, and **f**) are based on donors in Pediatric Longitudinal 1 dataset at: birth (*n* = 25), 3 months (*n* = 22), 6 months (*n* = 23), 1 year (*n *= 25), 1.5 years (*n* = 22), 2 years (*n* = 20), and 3 years (*n* = 23). **a** Boxplots of IgG concentration in children from birth-3 years of age overlaid on lines connecting exact measurements for each donor included. **b** Boxplots of the total number of VirScan peptide hits in the same donors overlaid on lines connecting exact values for each donor included. For boxplots in **a** and **b**, the center line indicates the median, box bounds indicate the 25th and 75th percentiles, whiskers extend to the smallest and largest values within 1.5× the interquartile range, and points beyond the whiskers indicate outliers. **c** Total number of maternal and endogenous hits for each donor in the data set. Maternal reactivities here are restricted to peptides recognized at birth with monotonically decreasing EBS values from birth to 1 year of age. **d** Inferred viral exposure (see Methods) in the first three years of life for three representative donors. New exposures are assigned to the earliest sampling interval meeting exposure criteria and distributed within that interval for visualization. Radii are varied to enhance clarity. **e** Stacked barplot of median number of hits in each age group colored according to the age at which a response to the peptide was first detected. **f** Stacked barplots displaying number of peptides newly targeted (top) or lost (bottom) relative to the previous time point colored in the same manner as panel e.

IgG concentrations were consistent with published values^31,32^ (Fig. 5a, Supplementary Data 6) and paralleled the trajectories of the total number of VirScan hits detected in the same samples (Fig. 5b).

Studying the antibody reactome in early life is complicated by the presence of both maternal and endogenous antibodies. When studying single samples from the first year of life, it is not possible to distinguish which responses are truly endogenous and could lend insight to early immune development. We found we could resolve this ambiguity by using longitudinal samples. We labeled peptide reactivities as maternal or endogenous based on the trajectories of their EBS values over time. Peptides with high baseline EBS values that declined were labeled as maternal, while peptides absent at birth but emerging later were labeled as endogenous (Fig. 5c). In line with established timelines of maternal antibody decay, we detect a rapid decrease in responses of maternal origin and expansion of endogenous responses during the first year of life (Fig. 5c), with most estimated half-lives of maternal antibody diversity falling below 100 days (Supplementary Table 1) in line with existing literature values^33–35^.

New viral exposures, acquired through natural infection and vaccination were a major driver of early-life reactome dynamics. Using 20 donors with follow-up from birth to at least 2.5 years of age, we estimated a mean of 22.15 ± 1.88 exposures (see Methods) in the first three years of life (Fig. 5d, Supplementary Fig. 6).

We next assessed persistence as a function of the age at which responses were first detected (Fig. 5e). We found that most of the endogenous antibodies produced in the first year of life do not persist beyond 6 months (Fig. 5e, f). The median number of responses that persist longer than 6 months increases for responses initiated at 1 year of age or later, but there is still a great deal of turnover even from ages two to three (Fig. 5f), consistent with observations that early-life B-cell responses tend to be short-lived and weaker^29^. Despite this turnover, children often later developed antibodies with many of the same endogenous specificities as their mothers for selected pathogens, including RSV (human orthopneumovirus), enteroviruses, some herpesviruses, and adenoviruses (Supplementary Fig. 7).

Virus-specific trajectories varied substantially across donors, although some pathogens, including enteroviruses and herpesviruses, showed more conserved patterns than others (Supplementary Figs. 8–10).

### Epitope-resolved profiling distinguishes maternal and endogenous antibody responses following early-life vaccination

Widespread vaccination has shifted early-life exposure to many pathogens from natural infection to controlled immunologic priming, making longitudinal birth cohorts a useful framework for studying how maternally derived antibodies shape early immune responses. In principle, sufficiently powered birth cohorts with detailed vaccination histories could enable quantitative dissection of maternal imprinting, antibody masking, and endogenous response maturation. Although the modest size of cohort C4 (Supplementary Data 7) precludes formal statistical inference, it nevertheless provides a proof-of-principle demonstration of how epitope-resolved seroprofiling can be used to reconstruct early-life vaccine response trajectories.

**Fig. 6:**
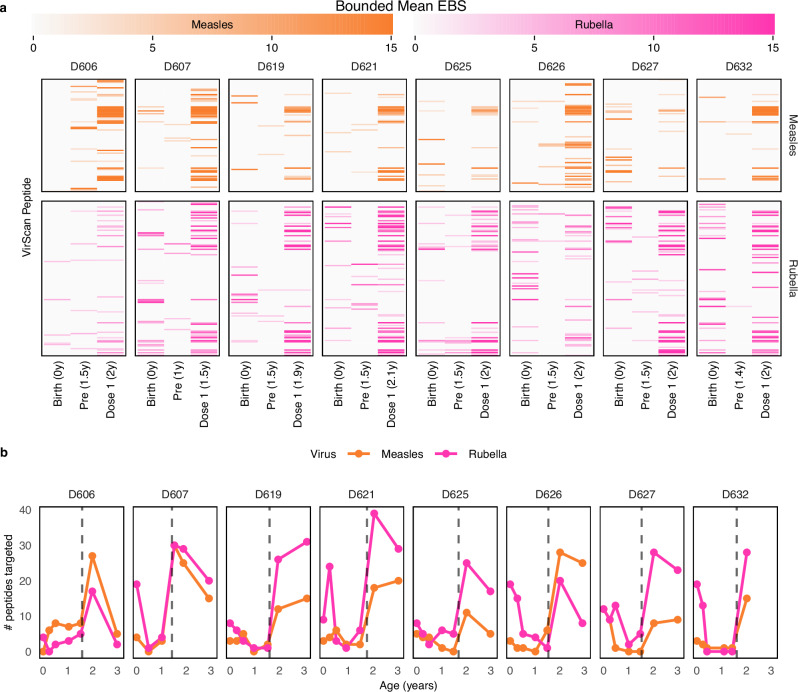
Longitudinal measles and rubella antibody responses before and after MMR vaccination in cohort C4. **a** Heatmap of peptide-level antibody reactivity to *Measles morbillivirus* and *Rubivirus rubellae* measured by VirScan. Rows represent individual viral peptides recognized at least once across the donor set; columns represent donor-specific key timepoints. For each donor, three classes of samples are shown: Birth, Pre (closest sample prior to first MMR dose at ≥6 months of age), and the first available sample after each recorded vaccine dose (e.g., Dose 1, Dose 2; mean age in years indicated on the x-axis). Color indicates bounded epitope binding score. **b** Number of recognized measles and rubella peptides over time for the same donors. Lines show the number of peptides targeted per sample (EBS ≥ 3.5), colored by virus (Measles, orange; Rubella, pink). Points indicate individual sampling timepoints. Dashed vertical lines denote the age at first recorded MMR vaccination.

MMR vaccination provides a relatively clean model of primary vaccine-induced immunity. In Finland, where measles and rubella have been eliminated since the late 1990s, vaccination occurs after maternal antibodies have waned^36^. Accordingly, pre-vaccination samples show only weak measles- and rubella-reactive signals, likely reflecting diminished maternal antibodies and low-level cross-reactivity given the fact that many of the peptides detected prior to vaccination are not detected post-vaccination (Fig. 6a). Following immunization, we observe robust expansion of virus-specific peptide recognition for both viruses with strong concordance among vaccinees (Fig. 6a). Although modest waning occurs in some donors, most retain broader virus-specific reactomes than before vaccination (Fig. 6b), consistent with effective primary vaccine-induced immunity.

Influenza vaccination occurred in a more complex epidemiologic context, with both seasonal (A(H1N1), A(H3N2), and B) and pandemic (2009 H1N1) strains administered in the setting of maternally derived antibodies and ongoing viral circulation. To contextualize antibody dynamics in early life, we align longitudinal epitope-resolved influenza hits with the timing of vaccinations and sample collection (Supplementary Fig. 11a). Across donors, endogenous profiles after vaccination frequently diverge from the maternal profiles observed at birth, both in the overall number of peptide hits assigned to each strain (Supplementary Fig. 11a) and when HA (hemagglutinin) and NA (neuraminidase) responses are considered separately (Supplementary Fig. 11b). Maternal profiles also exhibit marked inter-individual variation in strain specificity, consistent with differences in maternal exposure histories and potentially shaped by immunological imprinting phenomena such as antigenic seniority^37–39^. Although post-vaccination profiles show some convergence across children, particularly for NA (Supplementary Fig. 11b), substantial heterogeneity persists. This may reflect differences in age at vaccination, host genetics, and/or natural exposure to circulating influenza viruses during follow-up.

In contrast, vaccines administered during early infancy, such as poliovirus and rotavirus, are given in the presence of maternal antibodies and show more variable response patterns (Supplementary Fig. 12), providing a context in which maternal antibody masking may influence epitope selection and response magnitude.

Together, these examples illustrate how epitope-resolved seroprofiling can disentangle maternally derived and endogenous responses and characterize variability in early-life vaccine responses. Applied to larger cohorts, this approach could enable quantitative analysis of maternal antibody interference and vaccine response heterogeneity.

## Discussion

In this study, we define how antiviral serological repertoires are established, reshaped, and maintained across the human lifespan by profiling serum IgG binding across cross-sectional and longitudinal cohorts spanning infancy, childhood, and adulthood using VirScan. To our knowledge, this is the first lifespan-scale analysis to resolve antibody persistence at epitope resolution while distinguishing maternally derived from endogenous responses. These findings extend classical seroepidemiology beyond titers and serostatus to define generalizable principles governing the development and long-term maintenance of antiviral immune memory across the lifespan.

Early life was characterized by dense antigenic exposure and rapid remodeling of the antiviral antibody repertoire as maternally transferred IgG waned and endogenous responses accumulated. The natural exposures far outweigh those from vaccines. Pediatric repertoires were broader for shared exposures despite lower total IgG titers, whereas adult repertoires were narrower but highly stable over years to decades, generating reproducible individual-specific signatures that enabled highly accurate donor matching using Immunoprint. In this assay, breadth reflects linear epitope recognition and does not equate to B cell clonality, affinity maturation, or neutralization capacity, although each may partially correlate. Newborn and early-life humoral responses are also characterized by increased IgG polyreactivity^18–21^ and relatively elevated IgM and IgA titers^31,32^, which contribute to pathogen recognition and may shape subsequent IgG selection and consolidation. Because our profiling is restricted to IgG, these age-dependent differences in isotype composition and polyreactivity are not directly captured and may influence the apparent breadth observed in infancy.

The transition from breadth in early life to focused stability in adulthood correlates with progressive affinity maturation and selective consolidation of memory over time. Early-life B cell responses, characterized by reduced somatic hypermutation^14,18,21^ and germinal center responses that are quantitatively and functionally constrained relative to adults, including reduced selection stringency and altered differentiation into memory and plasma cell compartments^25,29^, may favor broader but less affinity-matured repertoires. In addition, early-life repertoires are enriched for polyreactive specificities^19,20,22^, which may further contribute to broader patterns of antigen recognition. Selection within germinal centers preferentially favors higher-affinity B cell clones for differentiation into long-lived plasma cells and memory compartments^40^, providing a plausible mechanism for the observed shift toward epitope focusing and long-term stability^7,8^. Thus, the transition to increased affinity maturation capacity may directly influence the establishment and persistence of antibody memory. Although epitope-scale serology cannot directly resolve these mechanisms, this framework provides a biologically plausible model for the age-dependent architecture observed. Moreover, seroreactivity does not distinguish primary infection from cross-reactive boosting, and exposure inferences should be interpreted accordingly^27,28^.

Durability varied systematically across viral families and was associated with both viral characteristics and features of the initial response. Reactivity to commonly recognized epitopes and stronger initial binding were linked to greater persistence, suggesting that early immunodominance and response magnitude influence long-term maintenance. The mechanisms responsible for differential durability across families may result from tropism or immune-escape mechanisms that distinguish viral families and remain to be explored. Because our peptide library captures linear determinants and not functional activity, these observations reflect epitope-binding persistence rather than protective immunity.

Longitudinal birth cohorts further demonstrated the value of epitope-resolved profiling for disentangling maternally derived from endogenous responses and for examining how vaccination timing and exposure history shape immune trajectories in early life. Integrating such profiling with detailed clinical metadata will be critical for translating epitope-scale serology into actionable insights for vaccine strategy and immune surveillance.

This study has limitations. Pediatric cohorts were modest in size, and sampling density differed across age groups. Analyses rely on thresholding and peptide display, precluding assessment of conformational epitopes, isotype composition, affinity maturation, and neutralizing function. Incomplete exposure histories limit causal attribution of reactivity changes. Future studies integrating epitope-scale profiling with clinical metadata, functional assays, and more frequent longitudinal sampling will refine estimates of antibody persistence and clarify how early-life immune maturation shapes long-term antiviral memory.

Together, these findings establish a detailed map of AAR development, diversity, and stability across the human lifespan. They highlight the dynamic, heterogeneous nature of early-life immunity, the remarkable stability and individuality of the adult reactome, and the value of longitudinal, high-resolution serology for defining principles of immune memory. These data further suggest that age-dependent differences in immune maturation shape both the breadth and durability of antibody responses, with potential implications for the timing and design of vaccination strategies. By generating an age-stratified atlas of antiviral reactome dynamics and a practical method to track them, this work lays the foundation for continuously updated immune maps that can inform immunization strategies, immune surveillance, and immunogen design. The approach is readily extensible to emerging pathogens, positioning epitope-scale serology as a potential tool for precision public health.

## Methods

### Study design

This is an observational study utilizing previously collected serum samples as described below. The objective of this study was to characterise AAR stability and lifespan dynamics at the virus and peptide levels. Sample size was determined by sample availability. Each serum sample was run on VirScan with two technical replicates to ensure that results were consistent between replicates. Samples with replicate correlations lower than 0.7 were excluded from analysis. We observed the specificities of antibody responses in all cases. Statistical analysis was used to evaluate stability of virus and peptide reactivities and age and virus-specific differences in the AAR.

### Human sera

Human specimens were obtained under a protocol approved by the local human research oversight committee, with informed written consent provided by the donors. Secondary use of all human serum samples for the purposes of this work was exempted by the Brigham and Women’s Hospital Institutional Review Board (protocol number 2013P001337). Samples originated from five cohorts (Table 1, Table 2). Cohort C1 is made up of donors admitted to large hospitals in Boston, Massachusetts, in March–April of 2020^11,41^. Samples in Cohort C2 originated from women enrolled in the NYUWHS between 1985 and 1991^42^. Samples in cohort C3 were collected from active-duty U.S. military personnel enlisted between 1993 and 2013. Samples from cohort C4 were collected from children in Finland enrolled in the DIABIMMUNE study at birth between 2008 and 2010 and followed for up to 3 years^43^. Cohort C5 includes children in the United States with genetic risk of type I diabetes who were enrolled between 2004 and 2010. All serum samples were stored in aliquots at –80 °C until use.

### VirScan

VirScan was performed following a previously published protocol^44^ using the original VirScan library^9^ or VirScan 2.0 library^26^ depending on when the sample was run. For the purpose of this study, only peptides of viral origin that were encoded identically in both the original VirScan and VirScan 2.0 libraries were considered for analysis. Analysis was further restricted to remove consideration of viruses with extremely rare human host pathogenicity or extremely low seroprevalence in the geographic regions study donors lived in. Peptides derived from the following viral species were considered in the study:

*Human mastadenovirus A, Human mastadenovirus B, Human mastadenovirus C, Human mastadenovirus D, Human mastadenovirus E, Betacoronavirus 1, Human coronavirus 229E, Human coronavirus HKU1, Human coronavirus NL63, Hepatitis B virus, Orthohepevirus A, Human alphaherpesvirus 1, Human alphaherpesvirus 2, Human alphaherpesvirus 3, Human betaherpesvirus 5, Human betaherpesvirus 6A, Human betaherpesvirus 6B, Human betaherpesvirus 7, Human gammaherpesvirus 4, Human gammaherpesvirus 8, Influenza A virus, Influenza B virus, Influenza C virus, Primate erythroparvovirus 1, Enterovirus A, Enterovirus B, Rhinovirus A, Rhinovirus B, Human orthopneumovirus, Rotavirus A, Rotavirus B, Alphapapillomavirus 10, Alphapapillomavirus 7, Alphapapillomavirus 9, Alphapapillomavirus 3, Alphapapillomavirus 6, Alphapapillomavirus 1, Human papillomavirus, Alphapapillomavirus 5, Alphapapillomavirus 8, Alphapapillomavirus 11, Mumps orthorubulavirus, Alphapapillomavirus 2, Betapapillomavirus 1, Measles morbillivirus, Human orthorubulavirus 2, Human respirovirus 3, Human respirovirus 1, Human orthorubulavirus 4, Alphapapillomavirus 4, Human papillomavirus type me180, Betapapillomavirus 3, Betapapillomavirus 2, Gammapapillomavirus 2, Alphapapillomavirus 13, Gammapapillomavirus 4, Human papillomavirus type 64, Gammapapillomavirus 1, Mupapillomavirus 2, Hepacivirus C, Vaccinia virus, Human picobirnavirus, Human polyomavirus 4, Human polyomavirus 3, Human polyomavirus 1, Rubivirus rubellae, Norwalk virus, Torque teno virus 3, Mamastrovirus 1, Human mastadenovirus F, West Nile virus, Enterovirus C, Aichivirus A, Sapporo virus, Molluscum contagiosum virus, Torque teno virus 1, Parechovirus A, Cardiovirus A, Hepatovirus A, Torque teno virus, Enterovirus D, Human metapneumovirus, Human polyomavirus 2, Gammapapillomavirus 3*.

### Data analysis

Read counts for each sample and peptide were transformed into Epitope Binding Signals (EBS), a previously described quantitative measure of antibody binding^26^. In this paper, we use a modified EBS designed to reduce noise. We require the EBS of a peptide across both technical replicates to exceed 2.5 in order to take the geometric mean of the replicate EBS values. If this condition is not met, the EBS for that sample and peptide is set to 0. In some cases, we bound the upper limit of EBS to 15 and call this a bounded EBS. Pairwise Pearson correlations in Fig. 3 were computed on bounded EBS values, either across the complete peptide library or restricted to peptides containing previously described public epitopes. We consider a peptide targeted by the donor or a “hit” if the EBS in both technical replicates is at least 3.5^9,44^.

#### UMAP analysis and sensitivity analysis

Unless otherwise noted, UMAP embeddings were generated from binary peptide hit matrices, where a hit was defined as an epitope with EBS ≥ 3.5 in both technical replicates. Default embeddings (Fig. 2a, Supplementary Fig. 1, Supplementary Fig. 2b, c) were computed using Euclidean distance with parameters n_neighbors = 15 and min_dist = 0.1.

To assess robustness of the observed age structure, we performed a sensitivity analysis across a grid of UMAP hyperparameters (n_neighbors ∈ {5, 15, 30, 50}, min_dist ∈ {0.001, 0.1, 0.5}, metric ∈ {euclidean, cosine}) and 10 random seeds per parameter combination. Embedding stability across seeds was quantified using silhouette scores and Procrustes alignment. Silhouette scores were computed for age-group separation within each embedding, and Procrustes similarity was used to assess concordance across embeddings generated with different random seeds.

Because embeddings generated with different hyperparameter settings can differ in geometry, Procrustes comparisons were restricted to embeddings computed with identical parameters. To further evaluate robustness to class imbalance, we repeated UMAP analyses on class-balanced subsets in which adult samples were downsampled to match the smallest pediatric group.

To confirm that observed age associations were not an artifact of dimensionality reduction, we performed PERMANOVA on Jaccard distances in the original high-dimensional space with permutations restricted within donors. An empirical null distribution was generated by permuting age labels 500 times.

#### Breadth of antibody response

Viral exposure for Fig. 2d was inferred for each sample and viral species using the following criteria: recognize at least one previously described public epitope for the virus, have at least one EBS ≥ 5 for the species, target at least two distinct epitopes for the virus. Distinct epitopes were defined as peptides not sharing a common substring of seven amino acids or more^9^. A Wilcoxon Rank Sum Test was only computed for each virus for which there were at least five adult samples and five pediatric samples with inferred viral exposure.

#### Immunoprint

We evaluated several possible methods for Immunoprint using a VirScan data set comprised of 567 samples from 431 unique individuals. We first computed six different pairwise Pearson correlation coefficients for the data set using raw epitope binding signals (Z scores), bounded Z scores (lower limit bounded to 0, upper limit bounded to 15), or hit data over either the complete viral library or a set of previously characterized public epitopes^6^.

We then used ROC analysis to evaluate the performance of Pearson correlation coefficients, computed over an array of different epitope subsets, as classification criteria for identifying samples from repeat donors and to arrive at the proposed method. For each of these epitope subset-specific correlation metrics, we determined the value of the correlation coefficient that maximized the sum of sensitivity and specificity and used that value as a threshold for identifying positive matches.

While many of the correlation measures evaluated performed with high sensitivity and specificity (Supplementary Fig. 3c, d), we discovered that using the Pearson correlation coefficient computed over public epitopes alone resulted in remarkable performance particularly considering this reduces the pool of epitopes analyzed to only about 3% of the library. While using the entire library performs marginally better, we have opted to use bounded Z scores over just the public epitopes in the Immunoprint method as it is less computationally intensive. We call this measure an *Immunoprint Score*, defined as the Pearson correlation of bounded epitope binding signals (EBS) computed across peptides containing previously described public epitopes.

Immunoprint was evaluated as a binary classifier distinguishing within- versus between-individual sample pairs based on pairwise similarity scores. We validated Immunoprint using an independent adult dataset comprising 178 samples, including 80 longitudinal samples from 40 donors with repeated sampling and 98 single samples from additional donors. Validation was performed using a resampling strategy in which samples were randomly partitioned into a database set (approximately two-thirds of samples) and a query set (approximately one-third of samples). Immunoprint scores were computed for all pairwise comparisons between query and database samples, and sample pairs were classified as originating from the same individual or from different individuals using a threshold selected to maximize the sum of sensitivity and specificity. Performance was evaluated across 5000 resampling iterations using standard classification metrics. Immunoprint achieved robust discrimination between within- and between-individual sample pairs, with very high classification accuracy in this independent validation cohort (Supplementary Fig. 3e).

#### Viral family-specific autocorrelation and peptide persistence

To quantify the stability of the antiviral reactome by viral family, we computed within-donor Pearson correlations of bounded EBS values across peptides within each viral family between a baseline sample and subsequent longitudinal samples, yielding family-specific correlation trajectories as a function of sampling interval. For group-level comparisons across ages, correlations were Fisher z–transformed and modeled using a semi-parametric mixed-effects model with a natural spline for sampling interval (df = 3) and a random intercept for donor (*z* ~ ns(years_between, df = 3) × family × age group + (1|donor)), restricting to sampling intervals ≤12 years; estimated marginal means were back-transformed to the correlation scale for visualization.

For peptide-level persistence analyses, we selected sample pairs separated by 4–6 years (prioritizing the pair closest to 5 years) and restricted to peptides with a baseline hit. We modeled persistence using logistic regression of follow-up hit status on baseline EBS (Hit_follow-up ~ EBS_baseline; binomial family). To summarize peptide-level retention within age groups, we computed for each peptide the fraction of baseline-positive donors remaining positive at follow-up and compared distributions for public versus private epitopes using two-sided Wilcoxon rank-sum tests. For visualization, peptides were limited to those targeted by ≥5 donors and ≥20% of the age group at baseline.

#### Downsampling analysis to assess effects of viral family size on repertoire correlation estimates

To assess whether differences in peptide library size across viral families influenced longitudinal repertoire correlation estimates, we performed a systematic downsampling analysis focused on adult donors. Adult donors were defined as individuals with longitudinal sampling across at least three time points spanning a minimum of 4 years. Ninety-six adult donors met these criteria and were included in the analysis.

VirScan enrichment Z-scores were merged with viral peptide annotations to assign each peptide to a viral family. Longitudinal repertoire correlation trajectories were generated using previously described methods, in which within-donor correlations between antibody repertoires at different time points were computed relative to an initial reference sample. Correlations were estimated separately for each viral family using only peptides belonging to that family.

Viral families differed substantially in peptide representation within the VirScan library, ranging from 1099 peptides (*Pneumoviridae*) to 17,072 peptides (*Herpesviridae*). To directly evaluate the impact of library size, we focused on families with sufficient representation to allow multiple downsampling depths (*Herpesviridae*, *Orthomyxoviridae*, and *Picornaviridae*). For each family, fixed numbers of peptides were randomly sampled without replacement from the full family-specific peptide set. Sampling depths spanned the minimum observed library size up to the maximum available for each family (*Herpesviridae*: 1000–17,000 peptides; *Orthomyxoviridae*: 1000–7500 peptides; *Picornaviridae*: 1000–5000 peptides).

For each sampling depth, downsampling was repeated for 200 independent iterations using a fixed random seed to ensure reproducibility. Within each iteration, repertoire correlations were recalculated using only the sampled peptides, and correlations were estimated for sample pairs separated by 2-year and 5-year intervals. Correlation values at zero years between identical samples were set to 1 and included to anchor trajectory models.

For each viral family, sampling depth, and time interval, we summarized results across iterations by computing the median estimated correlation and the 2.5th and 97.5th percentiles. These summaries were used for visualization and comparison across families and sampling depths. All analyses were performed in R using custom functions for trajectory generation and downsampling.

#### Early life viral exposure

We estimated how many new viral exposures children experienced in the first three years of life for pediatric donors in cohort C4 with follow-up from birth to at least 2.5 years of age (Fig. 5d, Supplementary Fig. 6). To do so, we calculated the number of new peptides per virus each donor targeted between the preceding and current time point for each for the duration of follow-up. From the resulting data, we calculated the maximum number of new peptides ever targeted per virus for each donor. We classified the age at exposure as the earliest age at which a donor targeted at least half the maximum number of new virus-specific peptides ever targeted by the donor, also requiring that a minimum of five new peptides be targeted. We further required the donor to target at least one previously defined^6,9^ public epitope for the given virus at the time of exposure.

We grouped the following closely related viruses together and labeled as follows for this analysis: Human adenovirus (Human mastadenovirus A-F), Human betaherpesvirus 6 (Human betaherpesvirus 6A-B), Enterovirus A/B (Enterovirus A-B), HSV ½ (Human alphaherpesvirus 1–2), Influenza A/B (Influenza A virus, Influenza B virus), Papillomavirus (members of the *papillomaviridae* family). For grouped viruses, we only counted one exposure per time interval if we detected exposure to multiple viruses within a group by the previous criteria. If the exposure criteria were met for a different member of the group at a later time point, it was included as an exposure at that time point. Only the earliest time point where the exposure criteria were met was counted for ungrouped viruses. For illustration purposes, we evenly distributed exposures across the time intervals in which they were detected.

#### Statistical analysis and graphics

All statistical analyses were performed in R (version 4.4.0)^45^. Figures were generated using R and Adobe Illustrator. The following packages were used for data analysis and figure generation: readr^46^, readxl^47^, dplyr^48^, tidyr^49^, ggplot2^50^, ggpubr^51^, cowplot^52^, grid, ggplotify^53^, umap^15,16^, pROC^54^, broom^55^, splines, emmeans^56^ and ComplexUpset^57^.

Permutational multivariate analysis of variance (PERMANOVA) was performed using 500 random permutations of age labels to assess the association between age group and reactome composition.

### Reporting summary

Further information on research design is available in the Nature Portfolio Reporting Summary linked to this article.

## Supplementary information

Supplementary Information

Peer Review file

Description of Additional Supplementary Files

Supplementary Data 1

Supplementary Data 2

Supplementary Data 3

Supplementary Data 4

Supplementary Data 5

Supplementary Data 6

Supplementary Data 7

Reporting Summary

## Data Availability

All data generated in this study are provided in the Supplementary Information (Supplementary Data 1–7). All reasonable requests for materials to the corresponding author will be fulfilled. The VirScan library is available from S.J.E. under a material transfer agreement with the Brigham and Women’s Hospital.

## Code availability

All custom code used to generate the figures and perform the analyses described in this study is available at GitHub: https://github.com/moriahm7942/Persistence-of-Memory.
