# Supplementary material for: Persistence of memory: lifespan dynamics of the human antiviral antibody reactome
Source: Nat Commun. 2026 Jun 22;17:7870. doi: 10.1038/s41467-026-74680-y (PMC13442855; doi:10.1038/s41467-026-74680-y)
Supplement: Supplementary file 1 [file 41467_2026_74680_MOESM11_ESM.pdf]

nature portfolio

Corresponding author(s): Stephen Elledge

Last updated by author(s): May 29, 2026

Reporting Summary

Nature Portfolio wishes to improve the reproducibility of the work that we publish. This form provides structure for consistency and transparency in reporting. For further information on Nature Portfolio policies, see our Editorial Policies and the Editorial Policy Checklist.

Statistics

For all statistical analyses, confirm that the following items are present in the figure legend, table legend, main text, or Methods section.

n/a	Confirmed	
☐	☒	The exact sample size (*n*) for each experimental group/condition, given as a discrete number and unit of measurement
☐	☒	A statement on whether measurements were taken from distinct samples or whether the same sample was measured repeatedly
☐	☒	The statistical test(s) used AND whether they are one- or two-sided<br>*Only common tests should be described solely by name; describe more complex techniques in the Methods section.*
☐	☒	A description of all covariates tested
☐	☒	A description of any assumptions or corrections, such as tests of normality and adjustment for multiple comparisons
☐	☒	A full description of the statistical parameters including central tendency (e.g. means) or other basic estimates (e.g. regression coefficient) AND variation (e.g. standard deviation) or associated estimates of uncertainty (e.g. confidence intervals)
☐	☒	For null hypothesis testing, the test statistic (e.g. *F*, *t*, *r*) with confidence intervals, effect sizes, degrees of freedom and *P* value noted<br>*Give P values as exact values whenever suitable.*
☒	☐	For Bayesian analysis, information on the choice of priors and Markov chain Monte Carlo settings
☒	☐	For hierarchical and complex designs, identification of the appropriate level for tests and full reporting of outcomes
☐	☒	Estimates of effect sizes (e.g. Cohen's *d*, Pearson's *r*), indicating how they were calculated

Our web collection on statistics for biologists contains articles on many of the points above.

Software and code

Policy information about availability of computer code

Data collection	Sequencing: Harvard Biopolymers Facility Genomics Core's pipeline for NextSeq500 and NextSeq 2000 data acquisition; 2017-2025
Data analysis	All data analysis was performed in R (version 4.4.0) with the following packages: broom (version 1.0.7), cluster (version 2.1.6), ComplexUpset (version 1.3.3), cowplot (version 1.1.3), cutpointr (version 1.2.0), data.table (version 1.15.4), dplyr (version 1.1.4), emmeans (version 1.11.2-8), forcats (version 1.0.0), ggplot2 (version 3.5.1), ggplotify (version 0.1.2), ggraph (version 2.2.2), ggpubr (version 0.6.0), grid (version 4.4.0), igraph (version 2.2.2), lme4 (version 1.1-35.3), patchwork (version 1.3.0), pheatmap (version 1.0.12), plotROC (version 2.3.1), pROC (version 1.18.5), purrr (version 1.0.4), readr (version 2.1.5), readxl (version 1.4.3), splines (version 4.4.0), stringi (version 1.8.4), stringr (version 1.5.1), tibble (version 3.2.1), tidyr (version 1.3.1), tidytext (version 0.4.3), UpSetR (version 1.4.0), uwot (version 0.2.4), gtable(version 0.3.6), here (version 1.0.1), vegan (version 2.7-2). Code used to generate any figure or perform any analysis is on Github at https://github.com/moriahm7942/Persistence-of-Memory/

For manuscripts utilizing custom algorithms or software that are central to the research but not yet described in published literature, software must be made available to editors and reviewers. We strongly encourage code deposition in a community repository (e.g. GitHub). See the Nature Portfolio guidelines for submitting code & software for further information.

Data

Policy information about availability of data

All manuscripts must include a data availability statement. This statement should provide the following information, where applicable:

- Accession codes, unique identifiers, or web links for publicly available datasets
- A description of any restrictions on data availability
- For clinical datasets or third party data, please ensure that the statement adheres to our policy

All data generated in this study are provided in the Supplementary Information (Supplementary Data 1-7). All reasonable requests for materials to the corresponding author will be fulfilled. The VirScan library is available from S.J.E. under a material transfer agreement with the Brigham and Women's Hospital.

Research involving human participants, their data, or biological material

Policy information about studies with human participants or human data. See also policy information about sex, gender (identity/presentation), and sexual orientation and race, ethnicity and racism.

Reporting on sex and gender	Donor sex is reported for all samples included in this paper. Sex was assigned by clinical staff conducting the original studies in which biospecimens were collected. Gender identity is not known or reported in this study. Sex was not analyzed as a variable or covariate.
Reporting on race, ethnicity, or other socially relevant groupings	Race and ethnicity are not reported in this study.
Population characteristics	Samples were obtained from donors ranging in age from birth to 99 years old. Only the age, sex, and country of sample collection for each donor is reported in this study.
Recruitment	Human specimens were obtained under a protocol approved by the local human research oversight committee, with informed written consent provided by the donors. Samples originated from five cohorts (Table S1, Table S2). All serum samples were stored in aliquots at –80°C until use.
Ethics oversight	Secondary use of all human serum samples for the purposes of this work was exempted by the Brigham and Women's Hospital Institutional Review Board (protocol number 2013P001337).

Note that full information on the approval of the study protocol must also be provided in the manuscript.

Field-specific reporting

Please select the one below that is the best fit for your research. If you are not sure, read the appropriate sections before making your selection.

☒ Life sciences ☐ Behavioural & social sciences ☐ Ecological, evolutionary & environmental sciences

For a reference copy of the document with all sections, see nature.com/documents/nr-reporting-summary-flat.pdf

Life sciences study design

All studies must disclose on these points even when the disclosure is negative.

Sample size	Sample size was determined by sample availability.
Data exclusions	Samples with replicate correlations lower than 0.7 were excluded from analysis .This criteria was pre-established as part of laboratory quality-control pipeline.
Replication	Each serum sample was run on VirScan with two technical replicates to ensure that results were consistent between replicates.
Randomization	Randomization is not applicable to this study because it is an observational study utilizing previously collected serum samples
Blinding	Blinding was not possible or relevant to our study.

Reporting for specific materials, systems and methods

We require information from authors about some types of materials, experimental systems and methods used in many studies. Here, indicate whether each material, system or method listed is relevant to your study. If you are not sure if a list item applies to your research, read the appropriate section before selecting a response.

Materials & experimental systems

n/a	Involved in the study
[x]	[ ] Antibodies
[x]	[ ] Eukaryotic cell lines
[x]	[ ] Palaeontology and archaeology
[x]	[ ] Animals and other organisms
[x]	[ ] Clinical data
[x]	[ ] Dual use research of concern
[x]	[ ] Plants

Methods

n/a	Involved in the study
[x]	[ ] ChIP-seq
[x]	[ ] Flow cytometry
[x]	[ ] MRI-based neuroimaging

Plants

Seed stocks	N/A
Novel plant genotypes	N/A
Authentication	N/A
