# Supplementary material for: Persistence of memory: lifespan dynamics of the human antiviral antibody reactome
Source: Nat Commun. 2026 Jun 22;17:7870. doi: 10.1038/s41467-026-74680-y (PMC13442855; doi:10.1038/s41467-026-74680-y)
Supplement: Supplementary file 2 [file 41467_2026_74680_MOESM1_ESM.pdf]

SUPPLEMENTAL FIGURES

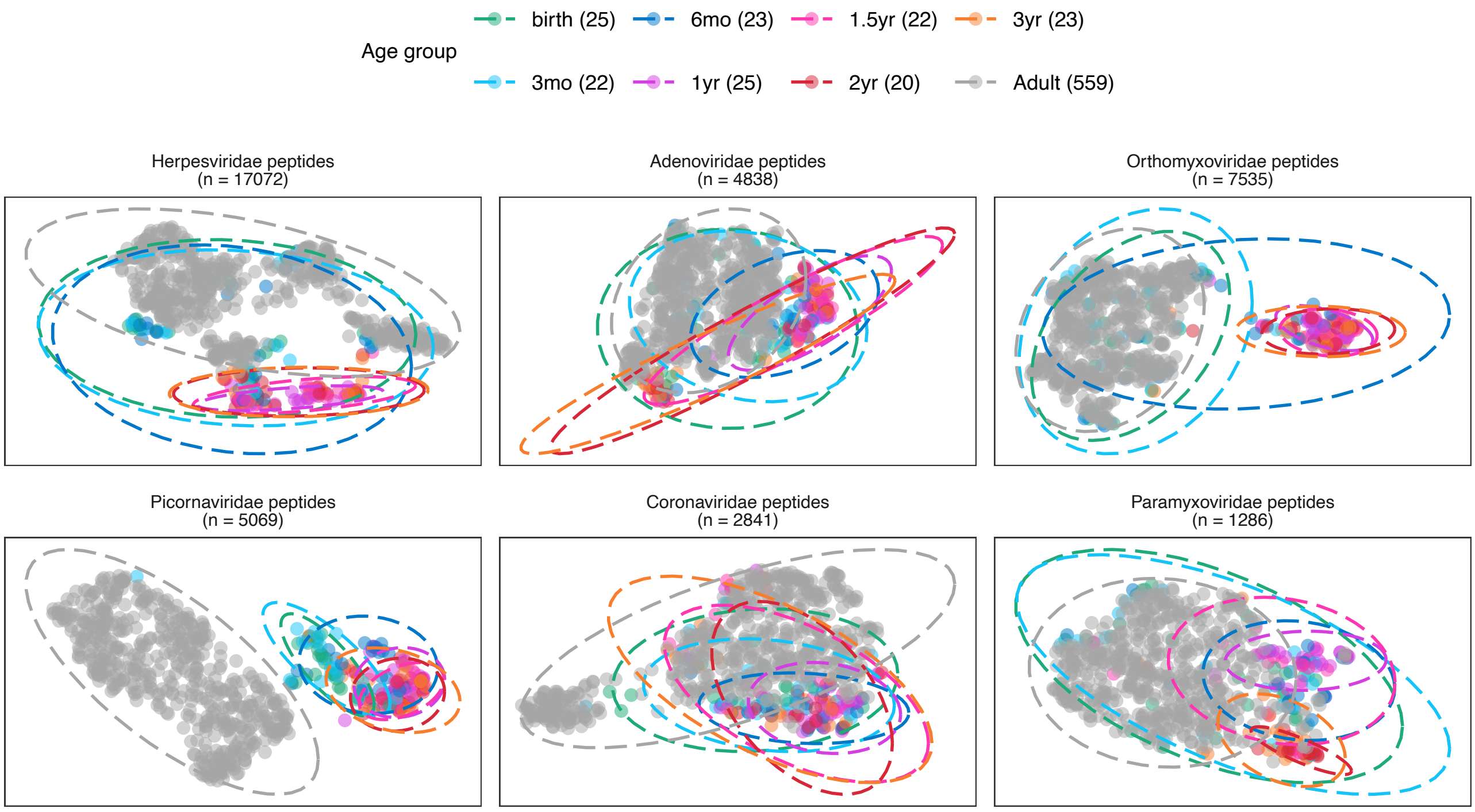

Fig. S1. Grouping of reactome responses by viral family and identity of seroprevalent peptides in children and adults. Output of UMAP dimension reduction plot generated using hit data by viral family. Biological replicates are distinct donor-timepoint samples; sample size for each age group is included in parentheses in the legend. Each dot is colored according to age of the donor at time of sample collection. Ellipses are drawn to enclose the area corresponding to a 95% confidence interval for the age group using a multivariate t-distribution.

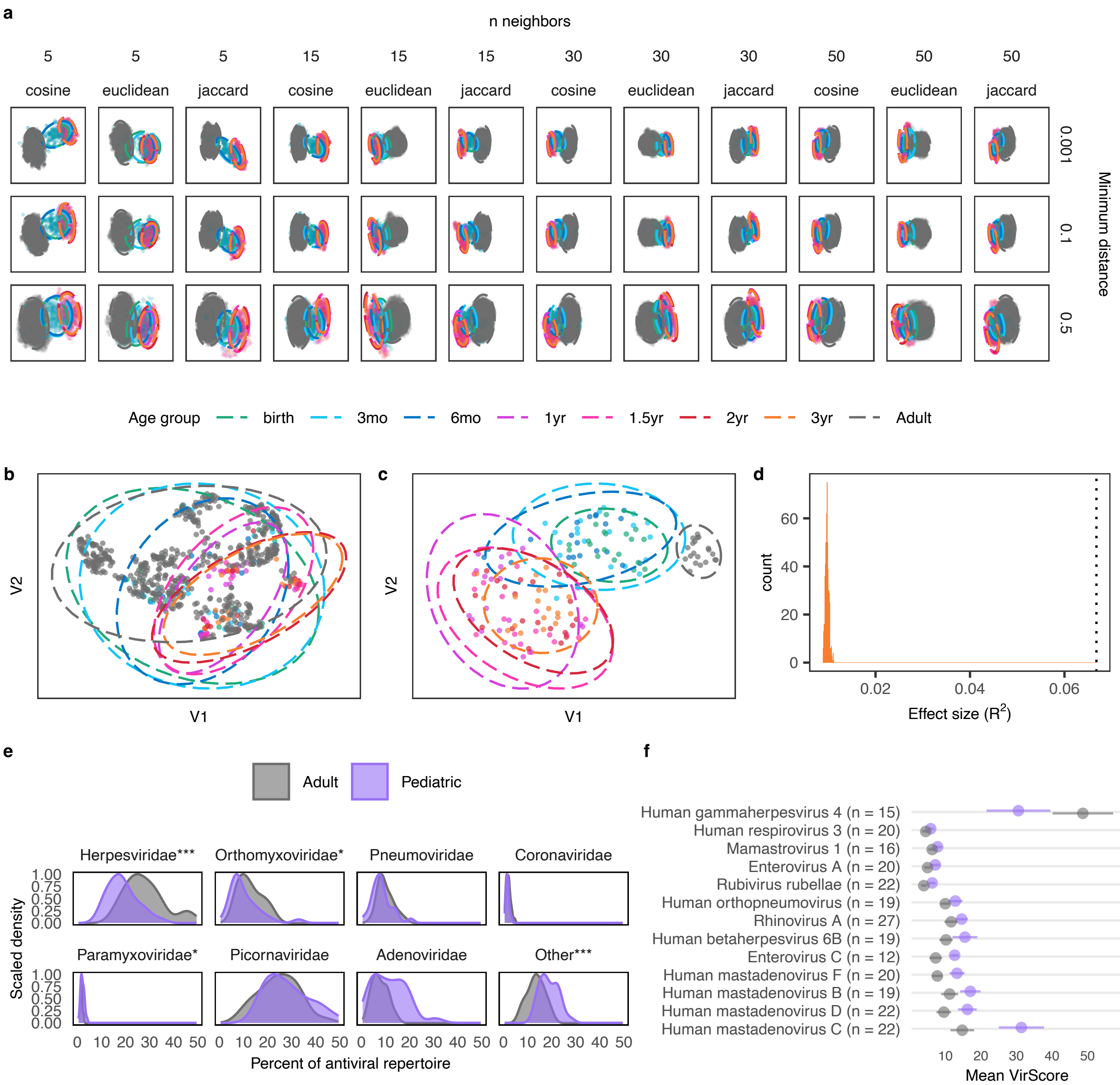

Fig. S2. Sensitivity analyses for UMAP and age-association robustness. Sample sizes for **(a-b)** were: birth (25), 3mo (22), 6mo (23), 1yr (25), 1.5yr (22), 2yr (20), 3yr (23), Adult (559). **(a)** UMAP embeddings of binary VirScan hit data across a grid of hyper-parameters (n_neighbors = {5, 10, 15, 30, 50}, min_dist={0.001, 0.1, 0.5}, metric ∈ {cosine, euclidean}) and 10 random seeds. Each point represents one donor-timepoint sample, colored by age group. Across hyperparameter settings and random seeds, age-group separation remained consistent (silhouette median = 0.155, IQR 0.031–0.280), and embeddings were highly concordant (Procrustes median t_0 = 0.91, IQR 0.84–0.98). **(b)** UMAP embeddings of VirScores colored by age group. Ellipses are drawn to enclose the area corresponding to a 95% confidence interval for the age group using a multivariate t-distribution. **(c)** UMAP embeddings of binary hit data generated from a class-balanced subset in which the number of adult samples was down sampled to match the smallest pediatric group (n=20). Ellipses are drawn to enclose the area corresponding to a 95% confidence interval for the age group using a multivariate t-distribution. **(d)** Empirical null distribution of PERMANOVA R^2 values obtained from 500 random label permutations (orange histogram) compared to the R^2 from the true age labels (black dotted line). **(e)** Distributions of percent reactome composition by viral family according to age group after downsampling the adult group to match the pediatric group. Biological replicates are distinct donors; adult and pediatric groups each included n = 27 donors. Statistical significance was assessed using two-sided Wilcoxon rank-sum tests comparing adults and children for each viral family. Asterisks denote nominal P values: *P < 0.05, **P < 0.01 and ***P < 0.001. **(f)** Mean VirScore ± 95% confidence interval among donors with inferred exposure after downsampling adults to match the pediatric group. Biological replicates are distinct donors; n values per age group are indicated after each virus name. Center values are means, and 95% confidence intervals were calculated using the t critical value and SEM. Statistical significance was assessed using two-sided Wilcoxon rank-sum tests; P values were unadjusted.

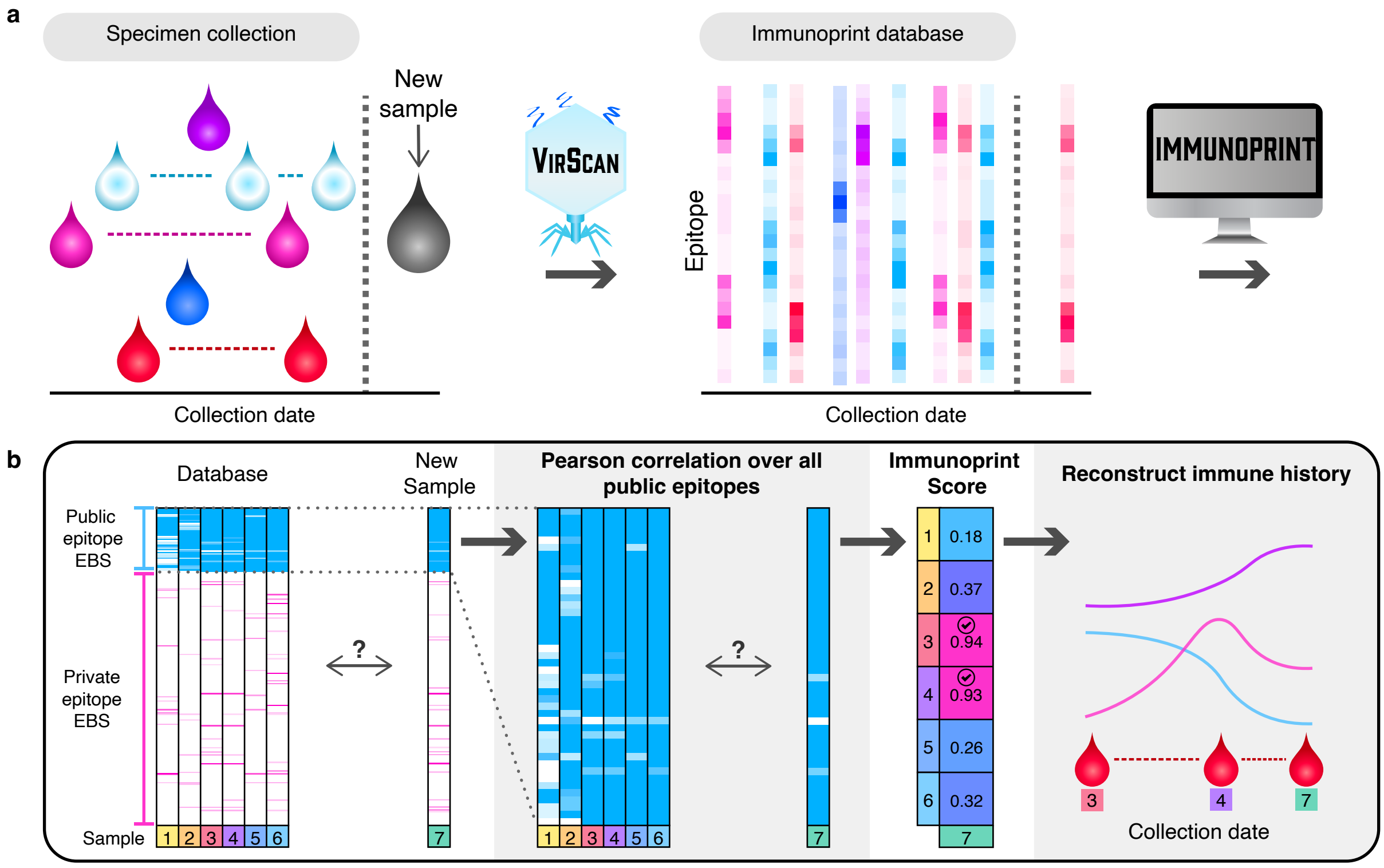

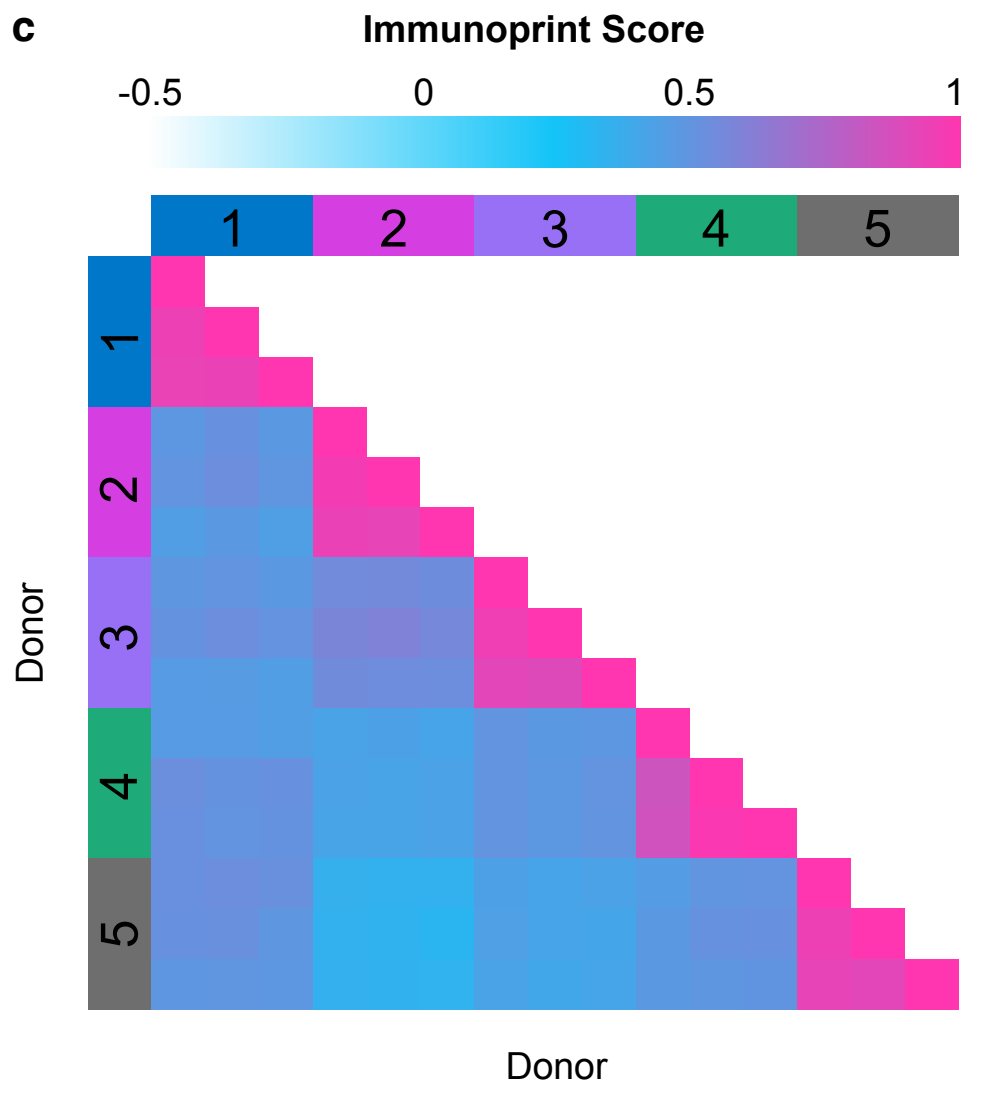

d

Data type	Peptide subset	Threshold	Accuracy (%)	FN	FP
Z (bounded)	All	0.66	99.99	4	0
Z (bounded)	Public	0.77	99.99	5	0
Z	All	0.81	99.97	20	18
Z	Public	0.88	99.97	38	7
Hit	All	0.52	99.98	24	5
Hit	Public	0.72	99.99	9	5

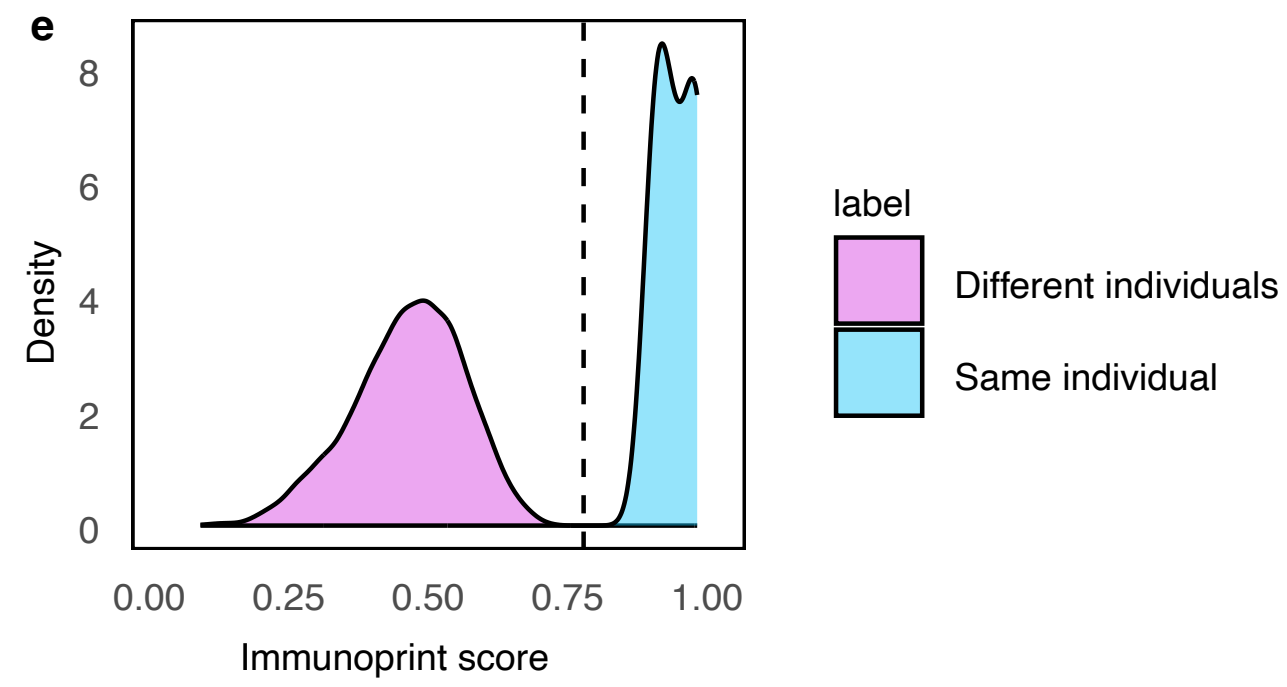

Fig. S3. Proposed Immunoprint quality control or surveillance workflow and performance. (a) Samples enter the surveillance system on a rolling basis. VirScan is performed and the results are compared to those in the existing database using Immunoprint. **(b)** Immunoprint compares the new sample's public reactome to the public reactomes of samples in the database using Pearson correlation. If the resulting Immunoprint Score exceeds a predetermined threshold, the sample pair is classified as an Immunoprint match and predicted to originate from the same donor. **(c)** Heatmap of Immunoprint Scores for comparisons between VirScan data for three longitudinal samples (collected at baseline, year 2, and year 6) from each of 5 donors. **(d)** Table of methods evaluated during Immunoprint development (see Methods). The final method is highlighted in blue. Although one method performed slightly better, the final method was selected based on simplicity and robustness. FP=False positive; FN=False negative. Thresholds were selected to maximize the sum of sensitivity and specificity. **(e)** Kernel density estimates of Immunoprint scores for sample pairs originating from the same individual (Same individual) or from different individuals (Different individuals) from a representative iteration of Immunoprint validation based on 177 samples from 137 distinct donors. Dotted line indicates Immunoprint classification threshold.

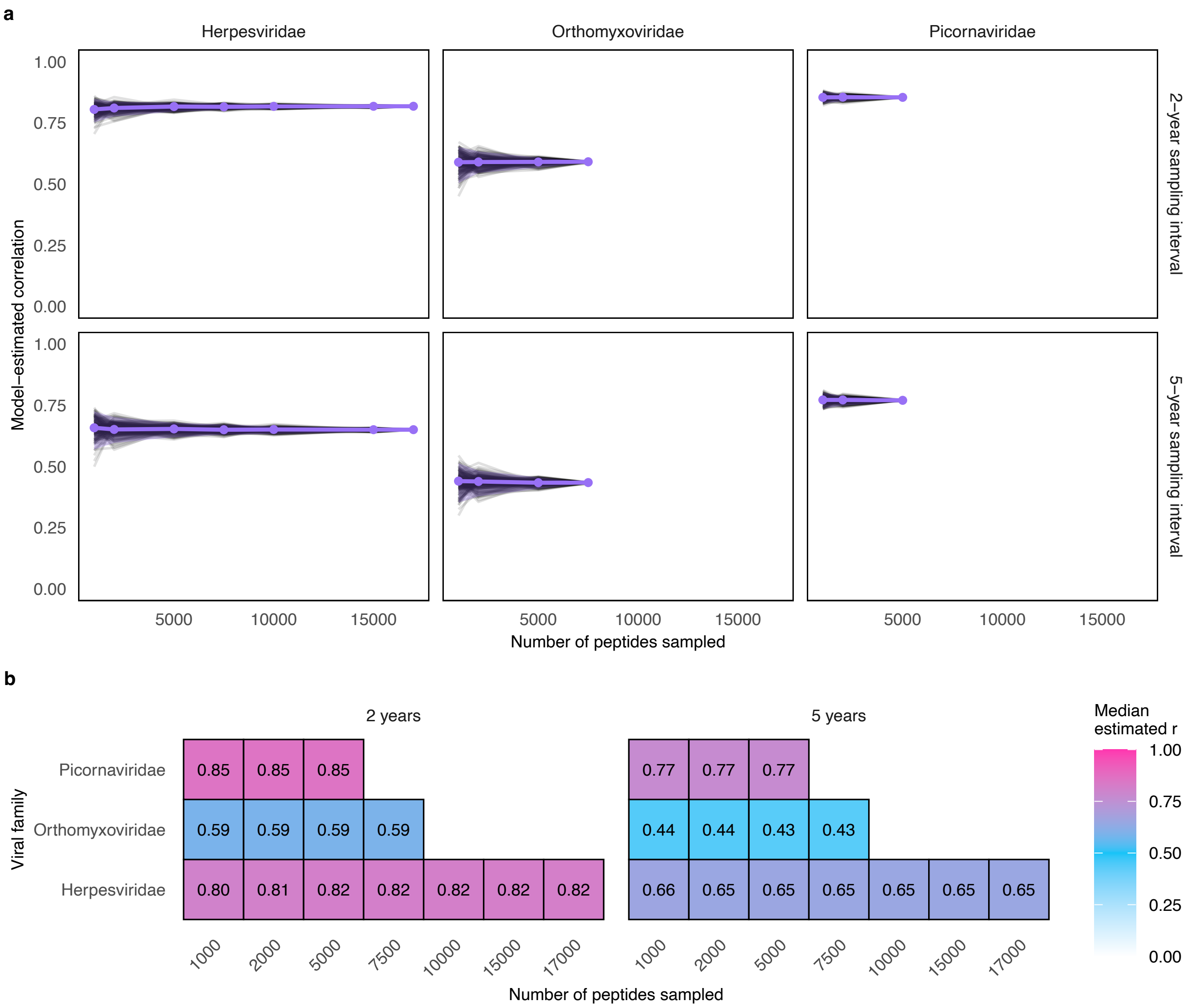

Fig. S4. Effect of viral peptide library size on longitudinal repertoire correlation estimates. To evaluate whether differences in peptide library representation across viral families influence longitudinal repertoire correlation estimates, we performed iterative downsampling of family-specific peptide libraries in adult donors (n = 96). Viral families varied widely in peptide coverage, and analyses focused on families with sufficient representation to support multiple sampling depths (*Herpesviridae*, *Orthomyxoviridae*, and *Picornaviridae*). Biological replicates are distinct adult donors. **(a)** Model-estimated repertoire correlation as a function of the number of peptides sampled, shown for 2-year and 5-year sampling intervals (rows) and viral family (columns). Thin lines represent individual downsampling iterations (n = 200 per sampling depth). Points and bold lines denote the median estimated correlation across iterations, and shaded regions indicate the 95% interval (2.5th–97.5th percentiles). **(b)** Heatmap summarizing the median estimated repertoire correlation for each viral family and peptide sampling depth at 2-year and 5-year sampling intervals. Values are annotated within each tile.

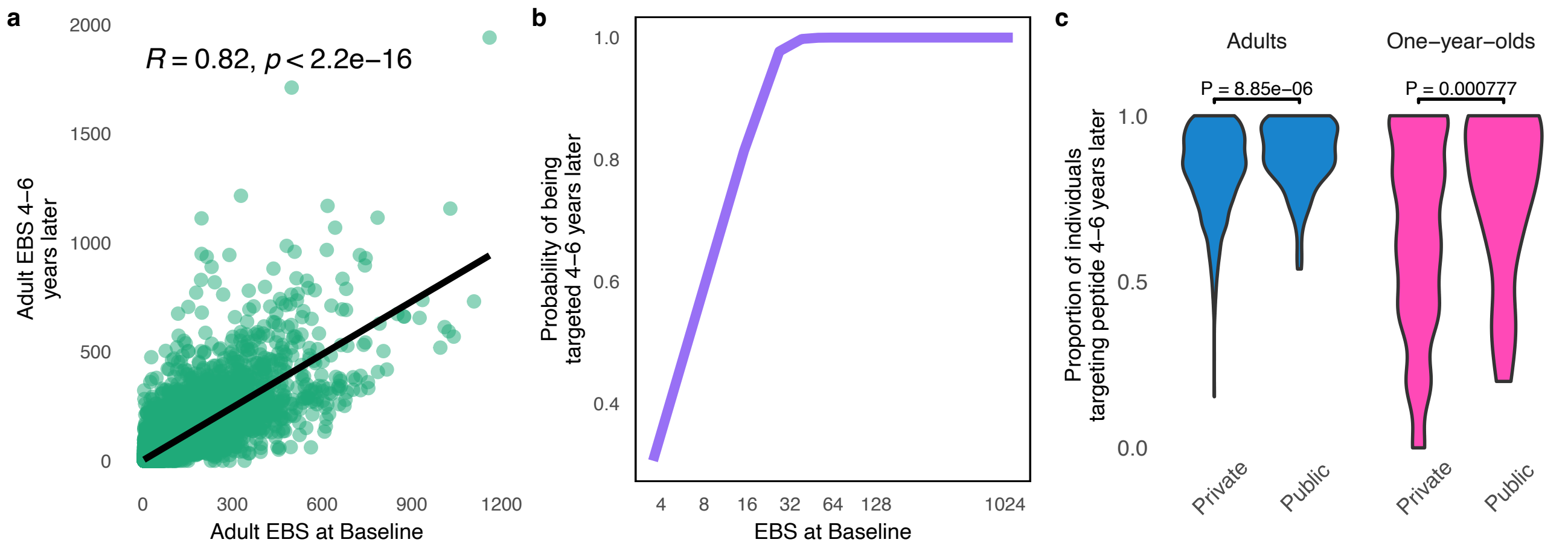

Fig. S5. Effect of initial EBS and immunodominance on persistence of response. (a) Scatter plot showing adult EBS at baseline and after 5 years, defined as a 4–6-year sampling interval. Each point represents one peptide recognized at baseline in one of 44 included adult longitudinal donors. **(b)** Predicted probability of a VirScan peptide targeted at baseline being targeted 5 years later according to baseline EBS. Probabilities were estimated from a logistic regression model fit to adult longitudinal data from 44 donors. **(c)** Violin plots showing the distribution of the proportion of peptides still targeted 5 years later across the entire library and among peptides containing previously characterized public epitopes for adults (n = 56 donors) and one-year-olds (n = 14 donors). Biological replicates are distinct donors. Statistical significance was assessed using two-sided Wilcoxon rank-sum tests, and exact P values are displayed above the comparisons for adults and one-year-olds.

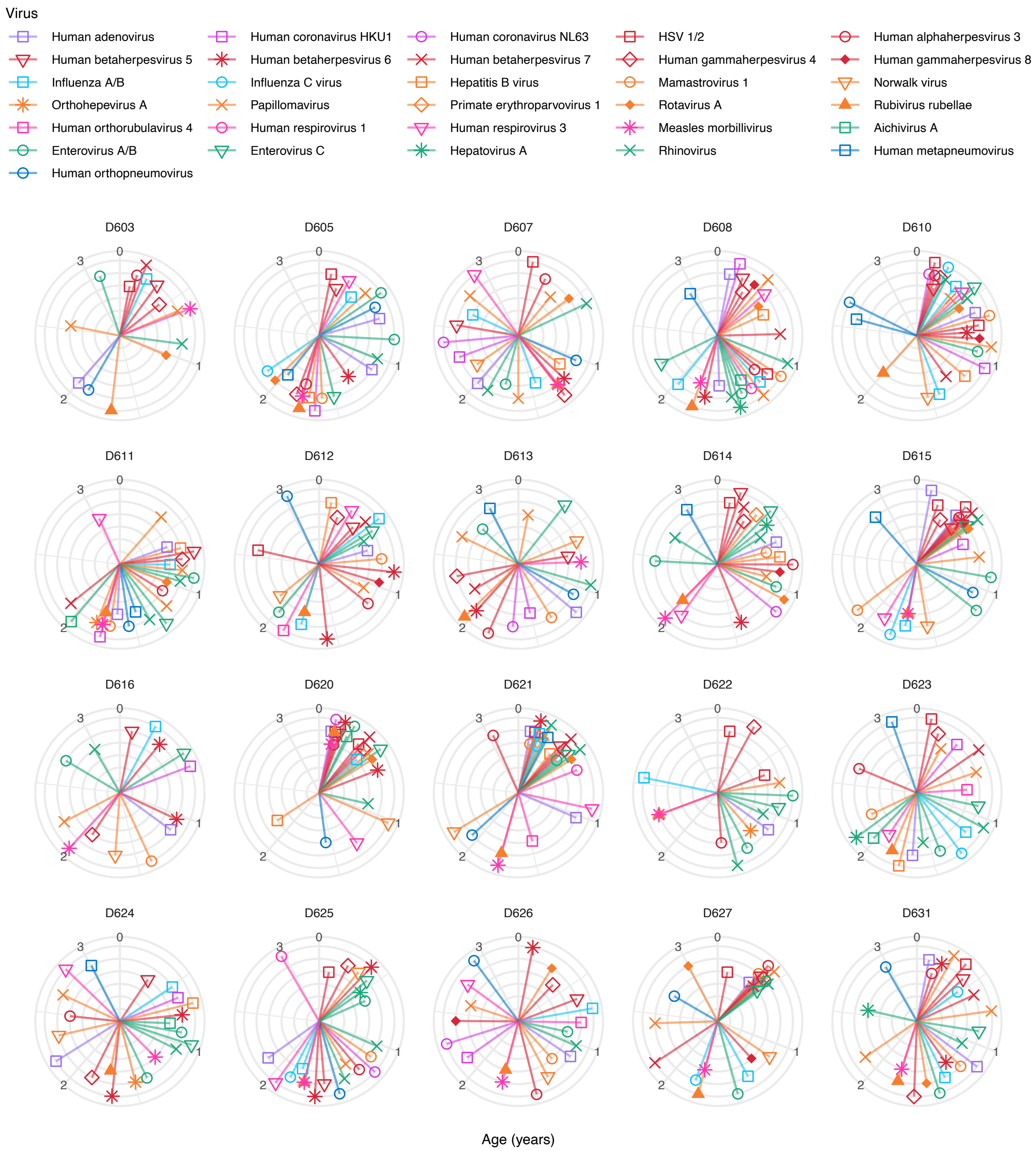

Fig. S6. Inferred viral exposure in the first three years of life. Inferred viral exposure (see Methods) in the first three years of life for 20 pediatric donors. New exposures are distributed across the sampling interval in which they were detected.

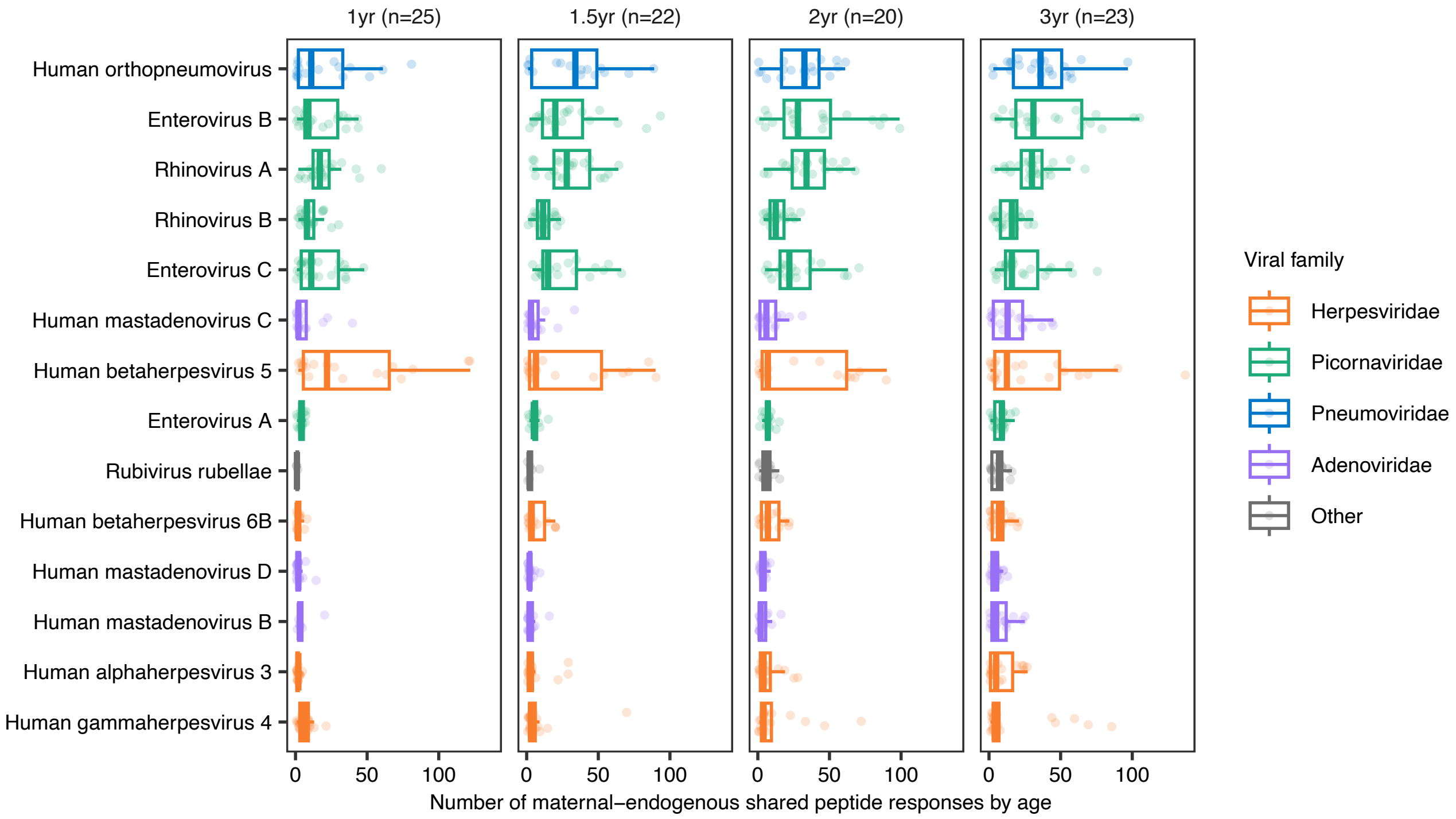

Fig. S7. Persistence of maternally shared antiviral antibody responses across early childhood. Number of viral peptides targeted by each child at the indicated ages that were also recognized in the maternal sample at the child's birth (maternal-overlapping peptide responses). Biological replicates are distinct children. For boxplots, the center line indicates the median, box bounds indicate the 25th and 75th percentiles, whiskers extend to the smallest and largest values within 1.5× the interquartile range, and points beyond the whiskers indicate outliers. Viral species are ordered by the median number of shared peptides at 3 years of age. Data are shown for the indicated time intervals (facet labels), with the number of children contributing to each interval provided in parentheses. Colors indicate viral family. Analyses are restricted to viral species with a median ≥5 shared peptides in at least one interval.

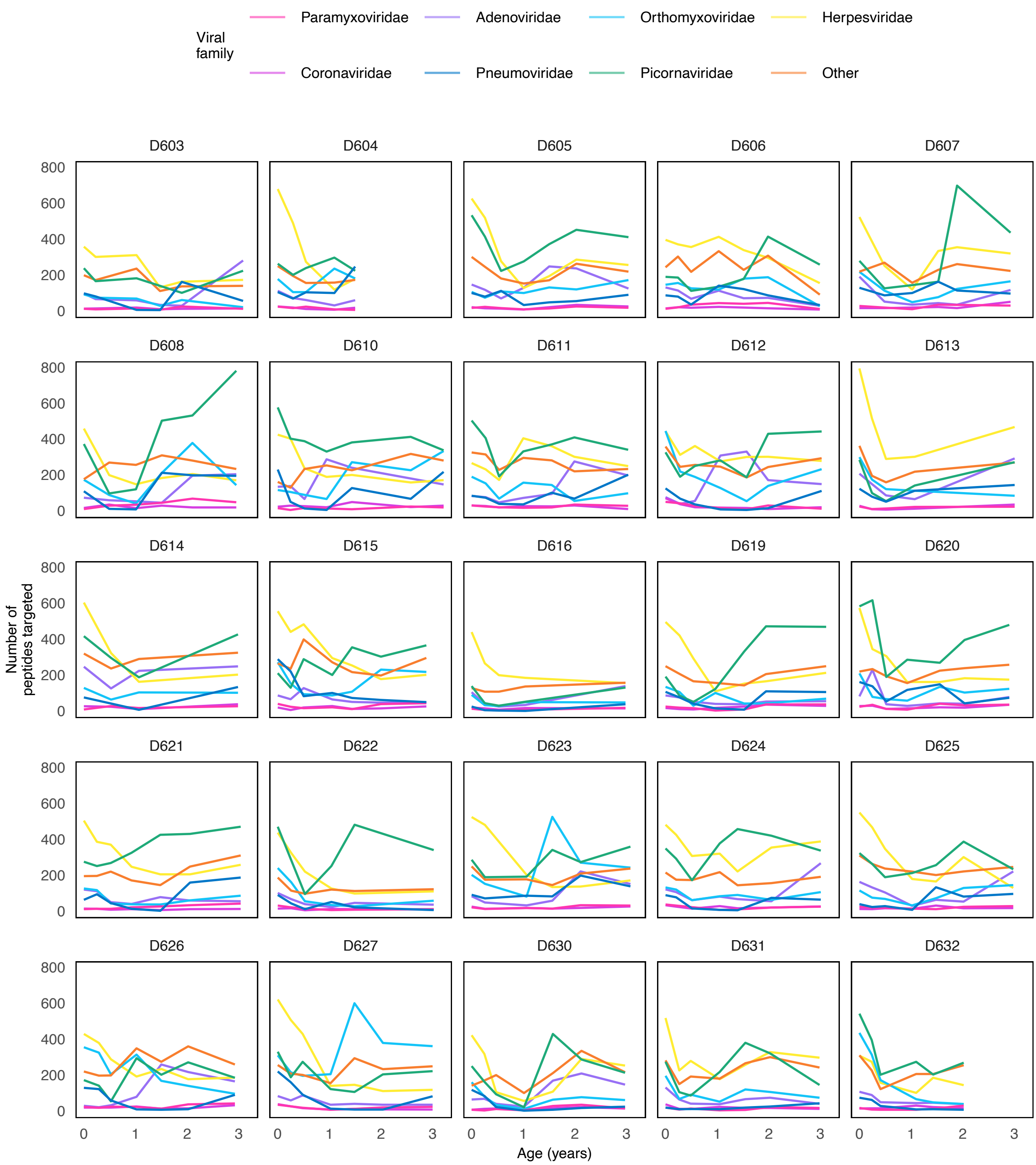

Fig. S8. Number of peptides targeted over time in the first three years of life. Each line connects the number of viral family-specific peptides targeted by a single donor from the Pediatric Longitudinal 1 dataset in the first three years of life. Biological replicates are distinct donors; n = 25 donors.

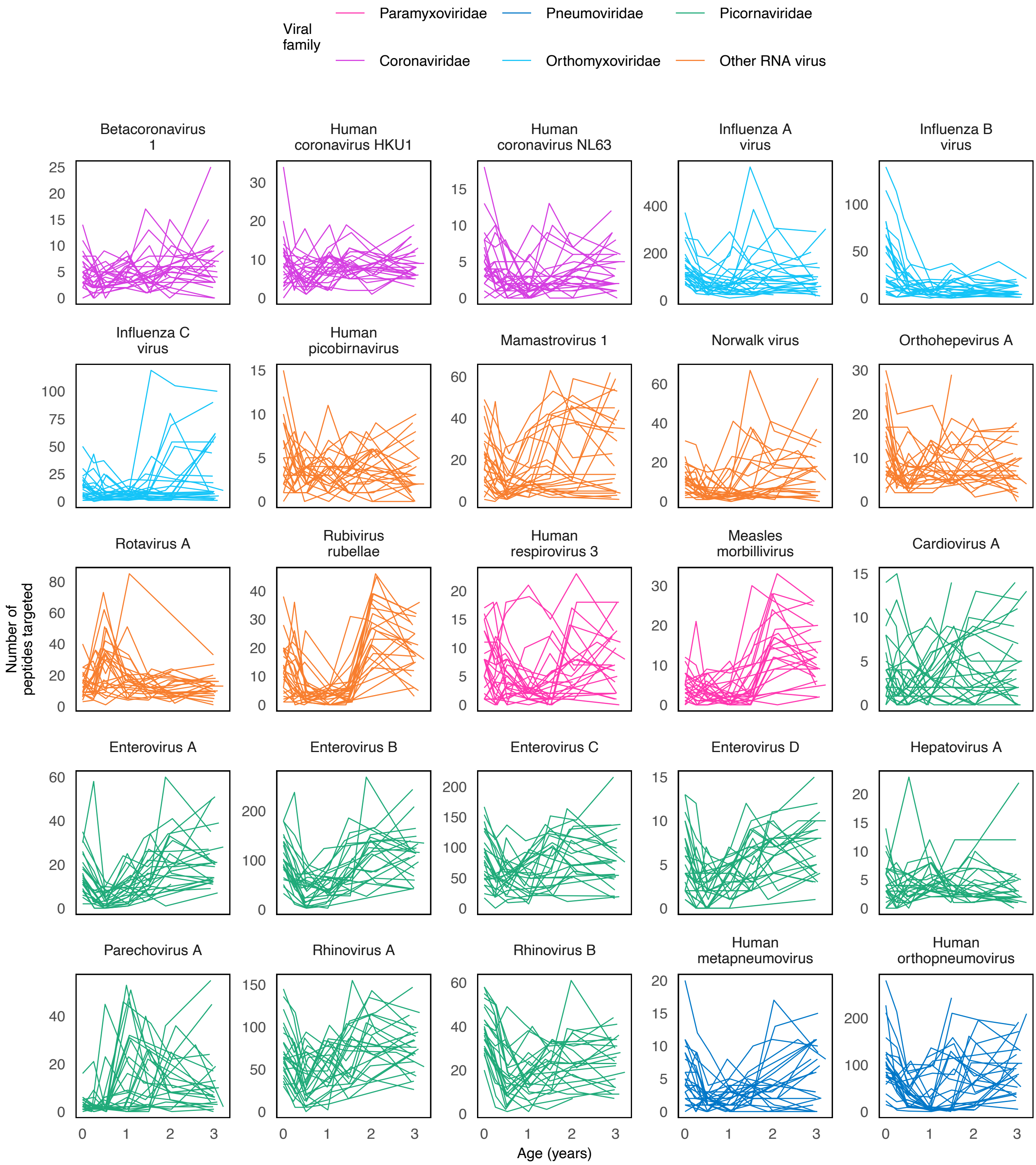

Fig. S9. Number of peptides targeted over time in the first three years of life for RNA viruses. Each line connects the number of virus-specific peptides targeted by a single donor from the Pediatric Longitudinal 1 dataset in the first three years of life for peptides derived from RNA viruses among the top 50 most frequently targeted viruses. Biological replicates are distinct donors; n = 25 donors.

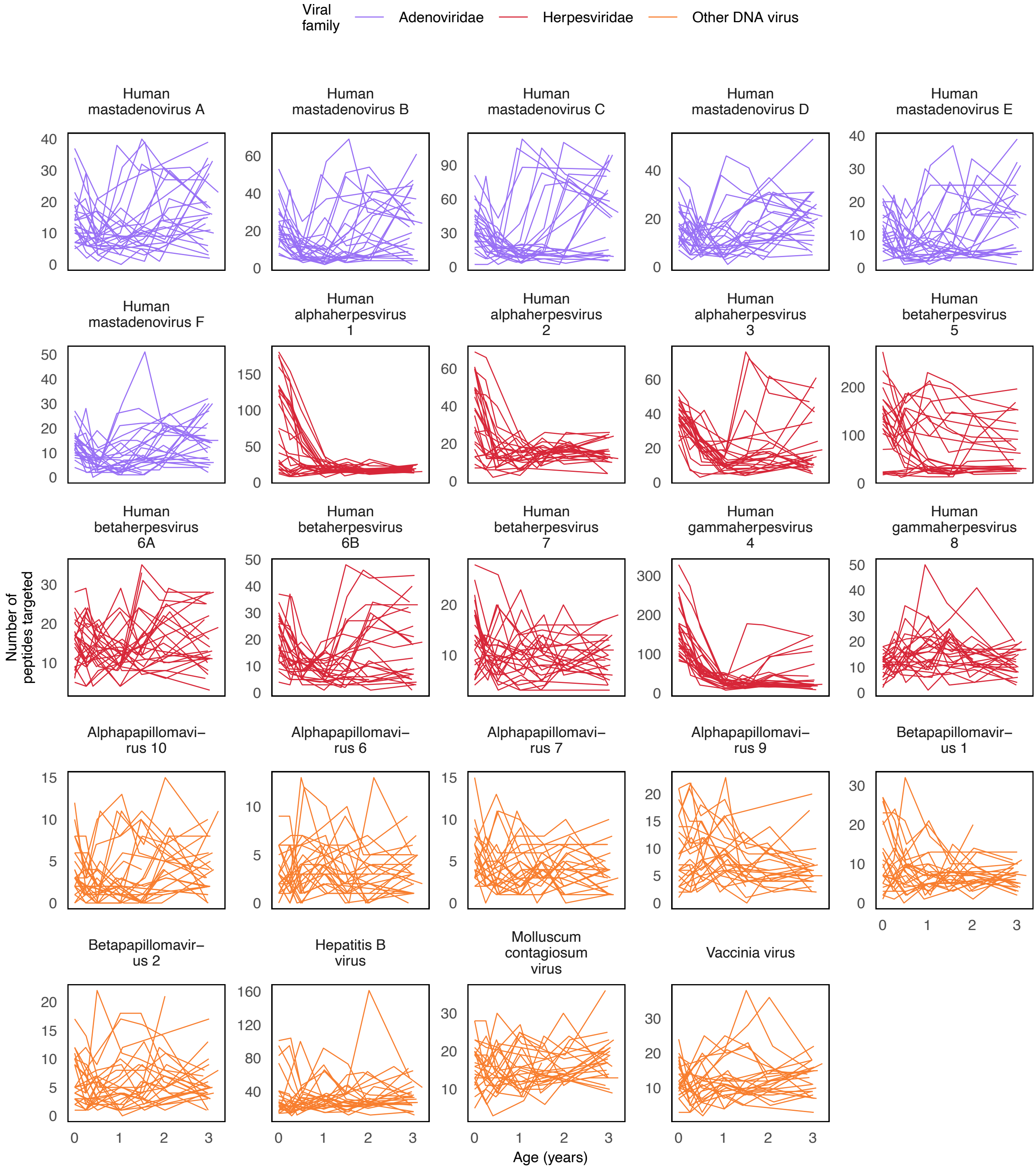

Fig. S10. Number of peptides targeted over time in the first three years of life for DNA viruses. Each line connects the number of virus-specific peptides targeted by a single donor from the Pediatric Longitudinal 1 dataset in the first three years of life for peptides derived from DNA viruses among the top 50 most frequently targeted viruses in the cohort. Biological replicates are distinct donors; n = 25 donors.

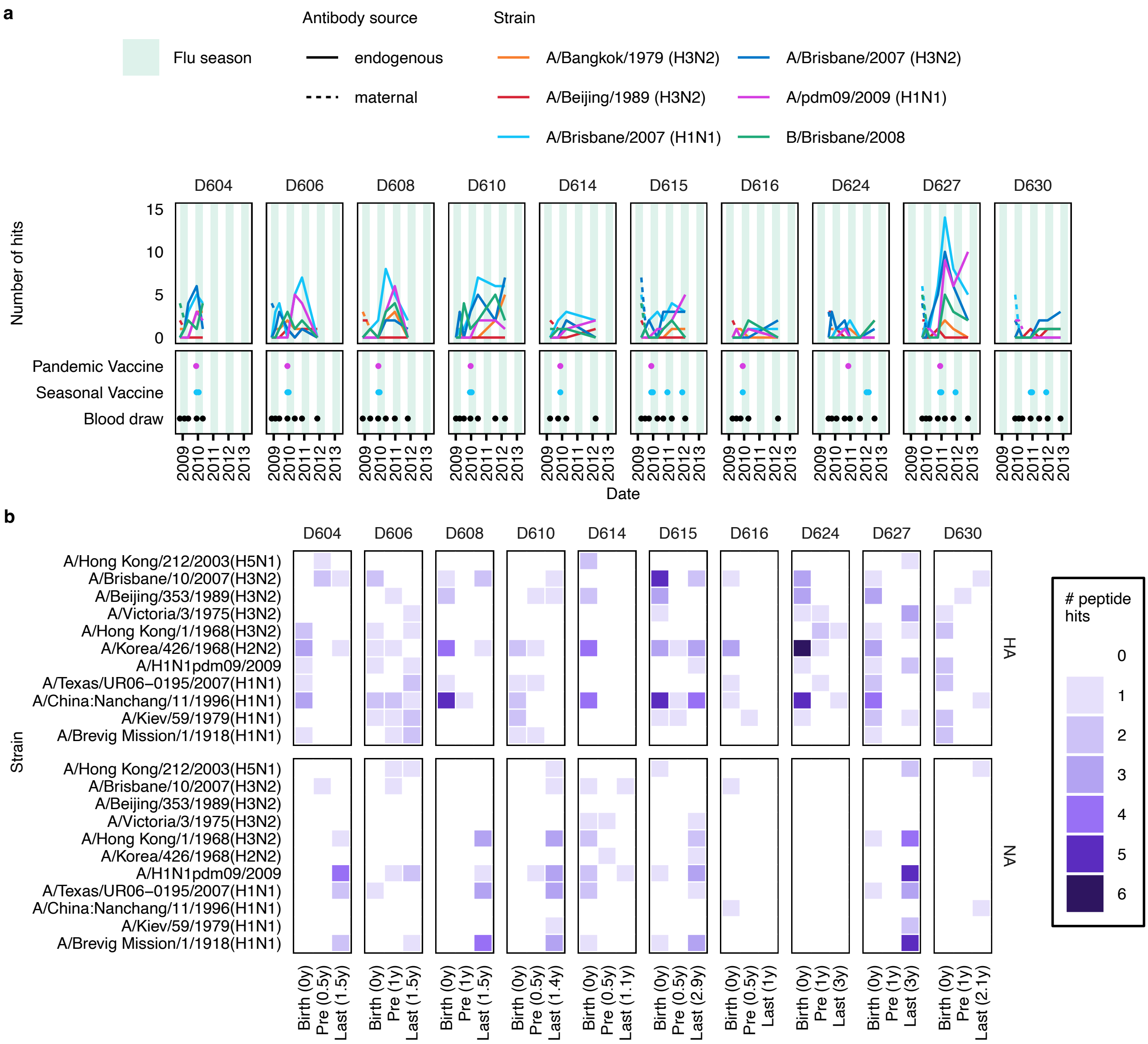

Fig. S11. Influenza vaccination responses to HA and NA are shaped by pre-existing antibody repertoires. Data are shown for n = 10 vaccinated donors. Biological replicates are distinct donors. **(a)** Longitudinal influenza peptide “hit” counts per donor across the sampling window. Lines show the number of influenza peptides called as hits (EBS ≥ 3.5) at each blood draw for each strain; line type indicates whether the response was classified as maternal-like (declining after birth) or endogenous (not maternal-like). Points below indicate blood draws and vaccination dates (seasonal vs pandemic influenza vaccine). Shaded regions mark influenza seasons. Pandemic 2009 H1N1 sequences were grouped as a single “A/pdm09/2009 (H1N1)” label. **(b)** Heatmap of the number of peptides hit (EBS ≥ 3.5) for hemagglutinin (HA) and neuraminidase (NA) from 11 antigenically distinct influenza strains at three key timepoints per donor: birth, pre-vaccination (closest sample prior to first influenza vaccination at age ≥ 9 months), and the first sample collected after the last influenza vaccine dose (“Last”). Rows are strains; columns are donor×timepoint; facets separate HA and NA and donors. Color encodes the number of peptides hit.

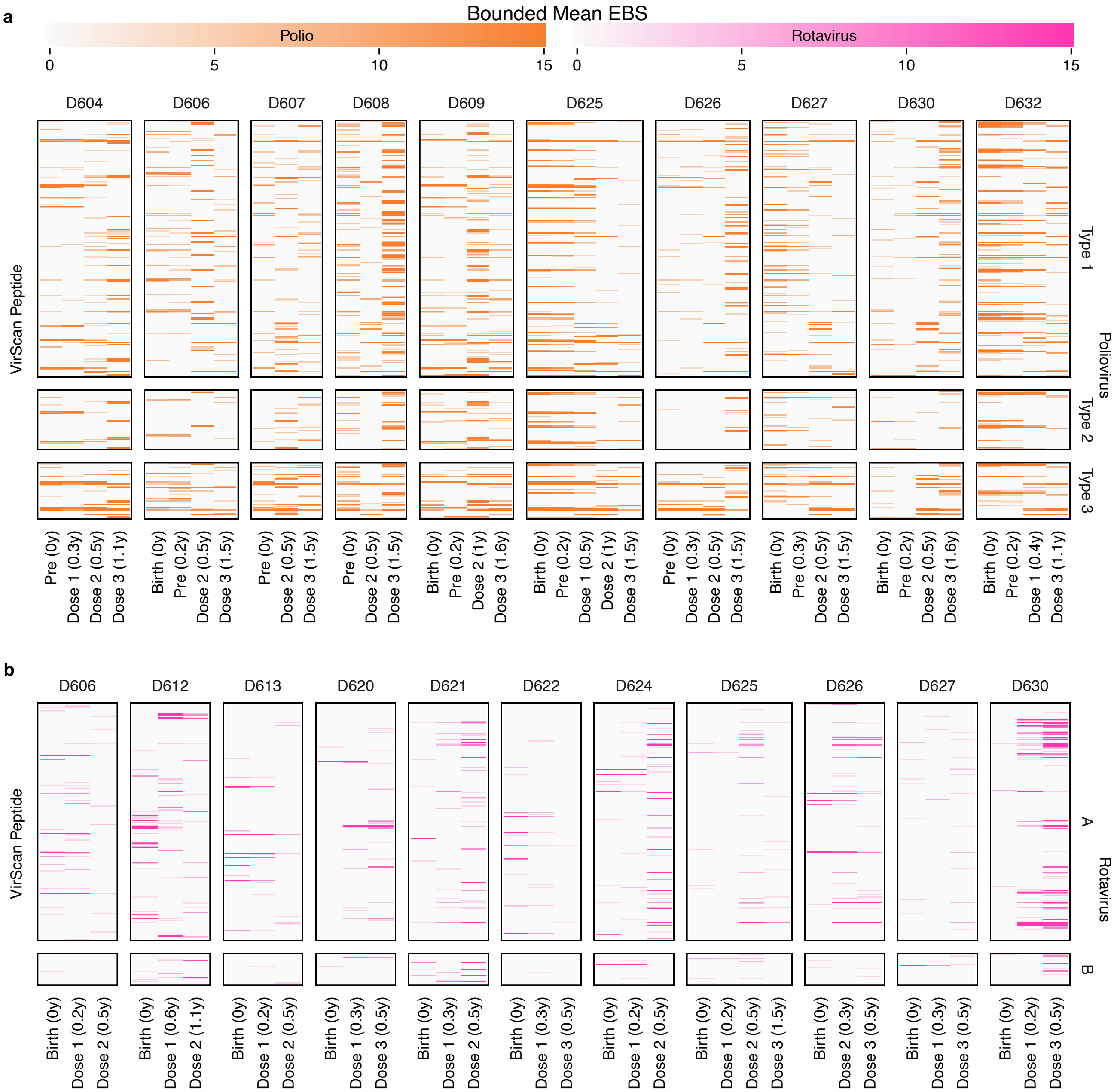

Fig. S12. Longitudinal poliovirus and rotavirus antibody responses across early-life vaccination. Heatmaps of antibody responses in vaccinated pediatric donors for **(a)** poliovirus types 1, 2, and 3 (n = 10 donors) and **(b)** Rotavirus A and Rotavirus B (n = 11 donors). Rows represent individual peptides recognized at least once in the set of plotted samples; columns represent Birth, Pre (closest sample prior to first vaccine dose), and the first available sample after each recorded vaccine dose, labeled by dose and age. Bounded Mean EBS is shown using color. Samples are faceted by donor.

SUPPLEMENTARY TABLES

Table S1. *Median and interquartile range of half-lives of maternal antibody diversity for groups of antigens*

Antigen category	*Median half-life (days)*	*IQR (days)*
Influenza A	49.3	31.7
Influenza B	68.8	37.8
Human orthopneumovirus (RSV)	63.6	33.1
Human adenoviruses	59.1	29.6
Human enteroviruses	52.5	31.1
Rubivirus rubellae (Rubella)	55.7	15.3
Measles morbillivirus	99.0	42.5
Human alphaherpesvirus 1 and 2 (HSV-1 and HSV-2)	59.1	31.8
Human gammaherpesvirus 4 (EBV)	60.9	20.0
Human betaherpesvirus 5 (CMV)	67.9	35.2
Other human herpesviruses	66.4	19.5
Human coronaviruses	45.4	19.5
Rotavirus	55.8	20.7
Other viruses	60.3	20.0
