# Supplementary material for: Persistence of memory: lifespan dynamics of the human antiviral antibody reactome
Source: Nat Commun. 2026 Jun 22;17:7870. doi: 10.1038/s41467-026-74680-y (PMC13442855; doi:10.1038/s41467-026-74680-y)
Supplement: Supplementary file 4 [file 41467_2026_74680_MOESM3_ESM.pdf]

Description of Additional Supplementary Files

File Name: Supplementary Data 1
Description: **Sample metadata.** Individual level metadata for all samples included. Values in the 'identifier' column correspond to column names containing hit and z score values for that sample in Supplementary Data 2 and Supplementary Data 3. Samples' inclusion status in the datasets described in Table S2 and Immunoprint development and validation are given in the last six columns of the file.

File Name: Supplementary Data 2
Description: **Epitope binding signals for cohort.** The id number of the Virscan peptide is given in the first column. The remaining columns contain the epitope binding signals (EBS) for each sample. These column names correspond to the 'Identifier' column in Supplementary Data 1. Negative EBS values have been set to 0.

File Name: Supplementary Data 3
Description: **Peptide hits for cohort.** The id number of the VirScan peptide is given in the first column. The remaining columns contain the hits for each case. These column names correspond to the 'Identifier' column in Supplementary Data 1. Hits are denoted with "1."

File Name: Supplementary Data 4
Description: **Library metadata.** The id number of the Virscan peptide is given in the first column. Entry and Entry name columns list the UniProtKB Entry and Entry name from which the peptide sequence was derived. Associated protein and gene names are given in the Protein names and Gene names columns. Amino acid sequences for the individual peptide and entry from which the fragment was derived are given by the peptide and Entry_sequence columns. The numerical NCBI taxonomy identifier and corresponding taxonomy information are provided in columns H to M. Public epitope status and family label used in the paper are given in public_epitope_2015 and family_label columns.

File Name: Supplementary Data 5
Description: **VirScores.** Species specific VirScores for each sample. 'Identifier' column can be used to link data to between supplemental files.

File Name: Supplementary Data 6
Description: **IgG concentrations for cohort C4.** IgG concentrations used in Figure 5. 'Identifier' column can be used to link data to between supplemental files.

File Name: Supplementary Data 7
Description: **Vaccination data for cohort C4.** 'Identifier' column can be used to link data to between supplemental files.
